# Exploring the links between SOS response, mutagenesis, and resistance during the recovery period

**DOI:** 10.1128/aac.01462-23

**Published:** 2024-03-27

**Authors:** Sreyashi Ghosh, Mehmet A. Orman

**Affiliations:** 1 Department of Chemical and Biomolecular Engineering, University of Houston, Houston, Texas, USA; Columbia University Irving Medical Center, New York, New York, USA

**Keywords:** antibiotic resistance, mutagenesis, DNA repair

## Abstract

Although the mechanistic connections between SOS-induced mutagenesis and antibiotic resistance are well established, our current understanding of the impact of SOS response levels, recovery durations, and transcription/translation activities on mutagenesis remains relatively limited. In this study, when bacterial cells were exposed to mutagens like ultraviolet light for defined time intervals, a compelling connection between the rate of mutagenesis and the RecA-mediated SOS response levels became evident. Our observations also indicate that mutagenesis primarily occurs during the subsequent recovery phase following the removal of the mutagenic agent. When transcription/translation was inhibited or energy molecules were depleted at the onset of treatment or during the early recovery phase, there was a noticeable decrease in SOS response activation and mutagenesis. However, targeting these processes later in the recovery phase does not have the same effect in reducing mutagenesis, suggesting that the timing of inhibiting transcription/translation or depleting energy molecules is crucial for their efficacy in reducing mutagenesis. Active transcription, translation, and energy availability within the framework of SOS response and DNA repair mechanisms appear to be conserved attributes, supported by their consistent manifestation across diverse conditions, including the use of distinct mutagens such as fluoroquinolones and various bacterial strains.

## INTRODUCTION

Mutagenesis is crucial from an evolutionary perspective as it introduces genetic diversity within populations, serving as a driving force for adaptation and survival. It creates the potential for advantageous traits that enhance the ability of an organism to survive in changing environments (1, 2). For instance, through mutations, bacteria can acquire genetic changes, conferring resistance to antimicrobial agents and facilitating survival and proliferation in their presence (3, 4). Bacterial species encounter diverse environmental disturbances that can harm their genetic material, posing a threat to genomic stability. These damaging factors include exposure to chemicals (such as fluoroquinolone antibiotics, mitomycin, bleomycin, aflatoxin B1), radiation [like ultraviolet (UV) light], or even byproducts of cellular metabolism [such as reactive oxygen species (ROS)] (5–11). Maintaining the effective repair of DNA lesions is vital for the survival of bacterial pathogens. In response to DNA damage, these organisms rely on the SOS response, which plays a crucial role in preserving genomic integrity (12). The bacterial SOS response network, controlled by a variety of regulatory proteins and enzymes, is highly conserved across different pathogens.

Upon DNA damage in bacteria, RecA molecules bind to single-stranded DNAs (ssDNAs), leading to the formation of a nucleoprotein filament. RecA, a crucial element in DNA repair, plays a pivotal role in multiple downstream processes by facilitating the cleavage of the transcriptional repressor LexA (13, 14). This cleavage event leads to the activation of the SOS regulon genes, encompassing a variety of proteins, such as DNA polymerases that enhance mutagenesis, recombinases that aid in the mobilization of antibiotic-resistance genes, and proteins that are involved in persistence and biofilm formation (15–19). The DNA repair process, which requires substantial energy, relies on specialized DNA polymerases like the translesion DNA polymerase (Pol) V (UmuC/D) (20). Pol V inserts random bases into the DNA strand opposite a lesion, facilitating the efficient bypass of the lesion by the replication fork (21). These lesions are not effectively passed through by the normal replicative DNA polymerase, Pol III. Also, Pol IV and II can contribute to mutagenesis in SOS-induced cells following antibiotic treatment (22). These SOS-induced repair mechanisms become crucial during the recovery phase after the removal of stress factors. To determine the role of quinolone-induced damage in antibiotic tolerance, two groups analyzed the SOS response in quinolone-treated *Escherichia coli* cells (23, 24). Their research revealed that antibiotic treatment induces a comparable level of damage in antibiotic-tolerant cells as it does in antibiotic-sensitive cells. Although antibiotic-tolerant cells can repair DNA damage, the repair machinery is not necessary until the recovery period (23, 25).

The primary objective of this study was to examine the effects of the SOS response on mutagenesis during recovery periods, utilizing highly mutagenic UV radiation and DNA-damaging conventional antibiotics. Despite the known links between SOS-induced mutagenesis and resistance mechanisms following the removal of mutagenic factors, there is a substantial knowledge gap regarding the role of the recovery period in shaping mutagenesis outcomes; the interplay of transcription, translation, and energy levels during mutagenesis recovery; and the development of strategic methods to mitigate mutagenesis. A comprehensive understanding of these aspects will offer valuable insights for the development of clinically pertinent therapeutic strategies against antibiotic resistance. Here, we opted for UV radiation as it is a widely employed mutagen that generates a broad spectrum of mutations without significant sequence preference (26, 27). Exposure to UV light leads to DNA lesions through various mechanisms, with the primary cause being the direct absorption of photons by nitrogenous bases, resulting in the formation of dimers between adjacent pyrimidines (28). Dimerization of pyrimidines halts DNA replication and generates gaps in ssDNA (28, 29). This triggers the SOS response, causing cell division arrest and the subsequent induction of DNA repair mechanisms. In this study, we observed that mutagenesis predominantly occurred during recovery after cells were exposed to UV, and inhibiting transcription/translation or depleting energy molecules at the start of treatment or during the early recovery phase reduced SOS response activation and mutagenesis. Inhibitors also prevented mutagenesis from fluoroquinolone antibiotics. Active transcription, translation, and the availability of energy are shown to be crucial for SOS response and DNA repair mechanisms, with the timing of inhibition being vital for disrupting DNA repair during the SOS response.

## RESULTS

### A correlation is evident between UV-induced SOS response and mutagenesis

**Fig 1:**
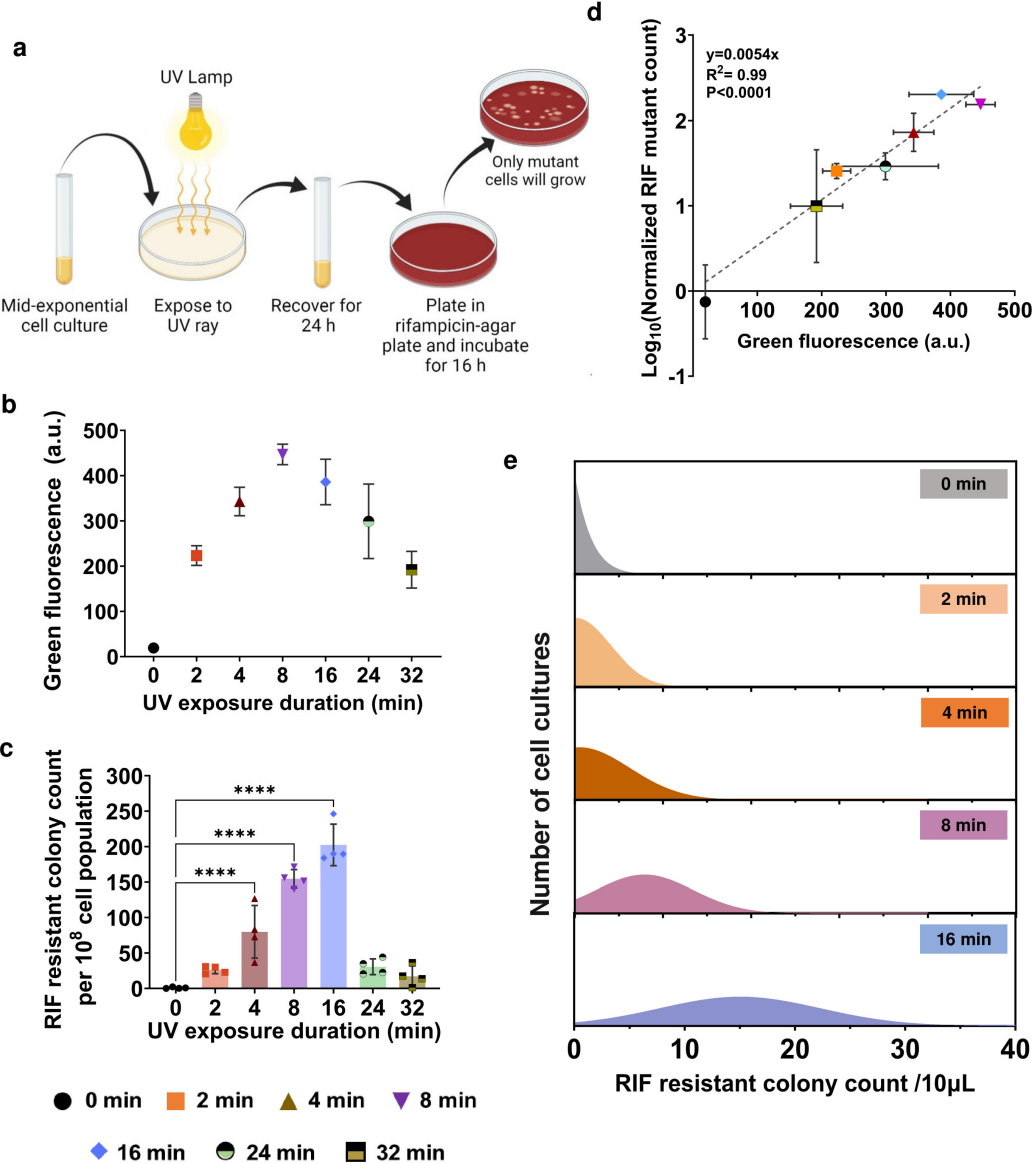
Correlation between UV-induced SOS response and mutagenesis. (**a**) Schematic representation of the methodology for inducing intracellular mutagenesis using UV as the mutagenic agent: Mid-exponential-phase *E. coli* MG1655 pUA66- P*_recA_-gfp* cells were exposed to UV for different durations and allowed to recover for 24 h. Following the recovery period, cells were plated on rifampicin (RIF)-agar plates to quantify the extent of mutagenesis. The schematic was made using BioRender software. (**b**) The expression profile of P*_recA_-gfp* at different UV exposure times after a 24-h recovery period. (**c**) Quantification of mutant cells reported as RIF-resistant colony count per 10^8^ cell population for different UV exposure times. (**d**) Correlation between *recA* expression level and normalized RIF-resistant mutants formed during different UV exposure durations. Simple linear regression analysis was performed to fit a straight line passing through the origin (y = 0.0054 x), R^2^ = 0.99 and *P* < 0.0001 (F-statistics). (**e**) Validation of the correlation between the UV-induced SOS response and mutagenesis levels utilizing a 96-well plate format. 288 independent cell cultures were similarly exposed to UV for varying times and then a 10 µL sample from each culture was plated on RIF agar plates after a 24-h recovery period. A frequency distribution was generated from the RIF-resistant colony data for each experimental condition and Gaussian distribution-based non-linear regression was employed to derive the probability distribution of RIF-resistant colony generation across various durations of UV exposure. For parts b, c and d: *n*  =  4; statistical analysis was performed using one-way ANOVA with Dunnett’s post-test, where ****P  <  0.0001; data corresponding to each time point represent mean value  ± standard deviation.

As the duration of UV exposure is pivotal in maximizing mutant yield, we first wanted to investigate the effect of UV exposure time on the RecA-induced SOS response. Given the rarity of mutagenic events, identifying the optimal UV exposure time that yields the highest mutation rate is crucial for studying mutagenesis during the recovery period in the subsequent sections. We utilized an *E. coli* strain harboring a RecA reporter plasmid (pUA66-P*_recA_-gfp*), where the expression of a green fluorescent protein (GFP) is controlled by the *recA* promoter (P*_recA_*). This system allows us to measure the activity of P*_recA_* and indirectly evaluate RecA expression levels based on GFP fluorescence (30, 31). In our study, we cultured *E. coli* MG1655 pUA66-P*_recA_-gfp* cells in 2 mL Luria Bertani (LB) medium in test tubes until they reached the mid-exponential phase with an optical density (OD_600_) at approximately ~0.5. Subsequently, the cells were transferred to Petri dishes, forming a thin film of cultures that increased the surface area for UV exposure. This film was then subjected to UV light (UVP ChemStudio, Analytik Jena) for various durations: 2, 4, 8, 16, 24, and 32 minutes (min). Following exposure, the cells were promptly transferred back to test tubes and cultured in a shaker for 24-hour (h) recovery (refer to the Materials and Methods section for details). Cultures that did not receive UV treatment served as controls. We opted for a longer recovery period, aligning with previous studies (32–34), recognizing the pivotal role of the recovery period in DNA repair mechanism activation (25) and mutagenesis (34). During the recovery phase, culture samples were collected to quantify colony forming units (CFU) using LB agar medium and assess *recA* expression using a plate reader (Fig. 1a). At t ~ 0 of the recovery period, we observed a decrease in CFU levels with increasing UV exposure time (Fig. S1a). For UV exposure durations of 4 and 8 min, a 10-fold decrease in CFU levels was evident, while 16 min of exposure resulted in a 100-fold decrease compared to the untreated control (Fig. S1a). Further prolonging the exposure time to 24 and 32 min led to a striking 10^5^-fold reduction in CFU levels (Fig. S1a). Surprisingly, after 15 min of recovery, a significant resurgence in CFU levels was observed specifically in cultures with longer UV exposure times, such as 24 and 32 min (Fig. S1a). We think that the reduction in CFU levels is not indicative of cell death, as it would be implausible for *E. coli* cells to exhibit a more than 1,000-fold increase in CFU levels within a mere 15-min period during recovery (considering the approximate doubling time of *E. coli*, which is around 25 min). This suggests that high levels of UV radiation rendered the UV-treated cells temporarily non-culturable for less than 15 min. When we monitored the expression of *recA* during the recovery phase, we observed that the fluorescence levels increased with longer UV exposure times, reaching their peak at 8 and 16 min of exposure, as illustrated in Fig. 1b (refer to Fig. S1b and c for the time-dependent profiles). However, further extending the UV exposure to 24 and 32 min led to a reduction in green fluorescence expression over time, compared to 8- and 16 min UV exposures (Fig. 1b). This reduction in *recA* expression aligns with the transient non-culturability observed in these treatment groups.

To investigate the effects of UV exposure times on mutagenesis, samples were collected from the recovery cultures after 24 h and spread onto an agar medium supplemented with 500 µg/mL rifampicin (RIF). These plates were then incubated to enumerate colonies that exhibited resistance to RIF, providing an indirect measurement of mutagenesis in the treatment groups, which is widely utilized in the literature (27, 34–36). The resulting data were normalized by calculating the number of RIF-resistant colonies per 10^8^ cell population (Fig. 1c). Although the number of cells in the treatment groups was similar at the 24-h time point of the recovery period (Fig. S1a), this normalization step was crucial to mitigate any inconsistencies that may arise due to variations in cell numbers. Our results demonstrated a consistent trend, where the number of RIF mutants increased gradually with longer exposure times, reaching the highest count in the treatment group exposed to UV for 16 min (Fig. 1c). However, the higher exposure times of 24 and 32 min resulted in a reduction in mutant formation (Fig. 1c). Notably, the profiles of RIF mutant levels and *recA* expression exhibited a similar pattern as UV exposure time varied. These findings demonstrate a strong correlation between the level of *recA* expression in SOS-induced cells and the rate of UV-induced mutagenesis (Fig. 1d). A similar trend in expression levels was also noted for *sulA* (which encodes a cell-division inhibitor) and *tisB* (which encodes a small membrane protein that inhibits ATP production)—both of which are well-studied SOS response genes (Fig. S2). This suggests that the observed trend is not specific to the *recA* gene; rather, it represents a general pattern in the SOS response.

### Extended UV exposure times in 96-well plates correlate with increased mutagenesis

It is important to note that our analysis was based on a limited number of culture samples (four biological replicates). Mutagenesis, a random event, still remains a rare process despite its induction by UV radiation. Even with the 16-min UV exposure, our observation revealed only a few hundred RIF-resistant mutants per 10^8^ cell population. To further validate the correlation between the UV-induced SOS response and mutagenesis levels, as well as to establish that this correlation is not an experimental artifact, we developed an experimental approach utilizing a 96-well plate format. In brief, 200 µL of cell cultures in each well of the plate, at mid-exponential growth phase (OD_600_ ~0.5), was exposed to UV for the specified durations (Fig. 1e; Fig. S3). Subsequently, the cells were allowed to recover for 24 h before being plated for the enumeration of RIF-resistant colonies (refer to Materials and Methods). This approach allowed us to substantially increase the sample size of our cultures. Our results demonstrated a notable alteration in the distribution of the variable (i.e., the level of RIF-resistant colonies) among different groups, with a pronounced skew towards “0” at lower UV exposure times, as expected due to lower anticipated mutagenesis rates under these conditions (Fig. 1e; Fig. S3). The 16-min UV exposure yielded a normal distribution with the highest mean value compared to the other groups (Fig. 1e; Fig. S3). In the preceding section, we demonstrated that extended UV exposure times (24 or 32 min) led to a reduction in the SOS response and mutagenesis in cells cultured in Petri dishes (Fig. 1a). However, it became evident that this phenomenon was not observed in cell cultures within 96-well plates, where more than 32 min were required for the inhibition of the SOS response. While this discrepancy may be attributed to distinct experimental conditions, we opted not to delve deeper into this matter, as we still detected an increase in RIF-resistant colony counts with increasing UV exposure times (up to 16 min) in these 96-well plates. Consequently, we will be utilizing 16-min UV treatments in the subsequent sections.

### A comparable correlation is also evident between H_2_O_2_-mediated SOS response and mutagenesis

The observed correlation between SOS response levels (as measured by *recA* expression) and mutagenesis should not be exclusive to UV exposure. To test this, we utilized hydrogen peroxide (H_2_O_2_) as an alternative mutagenic agent, known for its ability to directly cause DNA strand breaks, base modifications, and DNA adduct formation, potentially leading to a wide range of mutations (37, 38). To test the effects of this mutagenic agent, mid-exponential cells of *E. coli* MG1655 pUA66-P*_recA_-gfp* were subjected to varying concentrations of H_2_O_2_ (1.5 mM, 3 mM, 6 mM, 12 mM, 24 mM, and 32 mM) for 30 min (33). Following the treatment, the cells were thoroughly washed to eliminate hydrogen peroxide and allowed to recover for 24 h in fresh media. At specified intervals during the recovery phase, samples were collected for CFU, *recA* expression, and RIF-resistant colony measurements as described above. During the initial recovery phase, an increase in H_2_O_2_ concentration led to a slight decrease in CFU levels compared to the untreated control, with a more pronounced decrease observed at 24 mM and 32 mM H_2_O_2_ concentrations (Fig. S4a). However, unlike UV-treated cultures, this reduction in CFU levels was not transient in the H_2_O_2_-treated cultures. As the recovery time progressed, the cells gradually resumed proliferation, and after a 24-h recovery period, CFU counts became similar across all conditions (Fig. S4a). Regarding *recA* expression, we observed a gradual increase in green fluorescence over time as the concentration of H_2_O_2_ increased (Fig. S4b through d). The culture treated with 12 mM H_2_O_2_ exhibited the highest level of fluorescence (Fig. S4c and d). However, at higher concentrations of H_2_O_2_ (24 mM and 32 mM), there was a significant reduction in green fluorescence (Fig. S4c and d). Also, an incremental rise in the number of RIF-resistant colonies was observed with increasing H_2_O_2_ concentration, up to 24 mM. Notably, the culture treated with 12 and 24 mM H_2_O_2_ exhibited the highest number of RIF mutants, while 32 mM H_2_O_2_ significantly impeded mutant formation (Fig. S4b). Although the smaller number of bacterial cells surviving 32 mM H_2_O_2_ treatment may limit the likelihood of mutational events, this trend cannot be solely explained by the observed reduction in cell survival, as a growth deficiency was also observed in the 24 mM H_2_O_2_ condition. Despite the observed growth deficiencies, the trend between H_2_O_2_-induced SOS response and mutagenesis is still similar to that of UV-treated cultures.

### Inhibiting the expression or function of RecA chemically reduces mutagenesis

**Fig 2:**
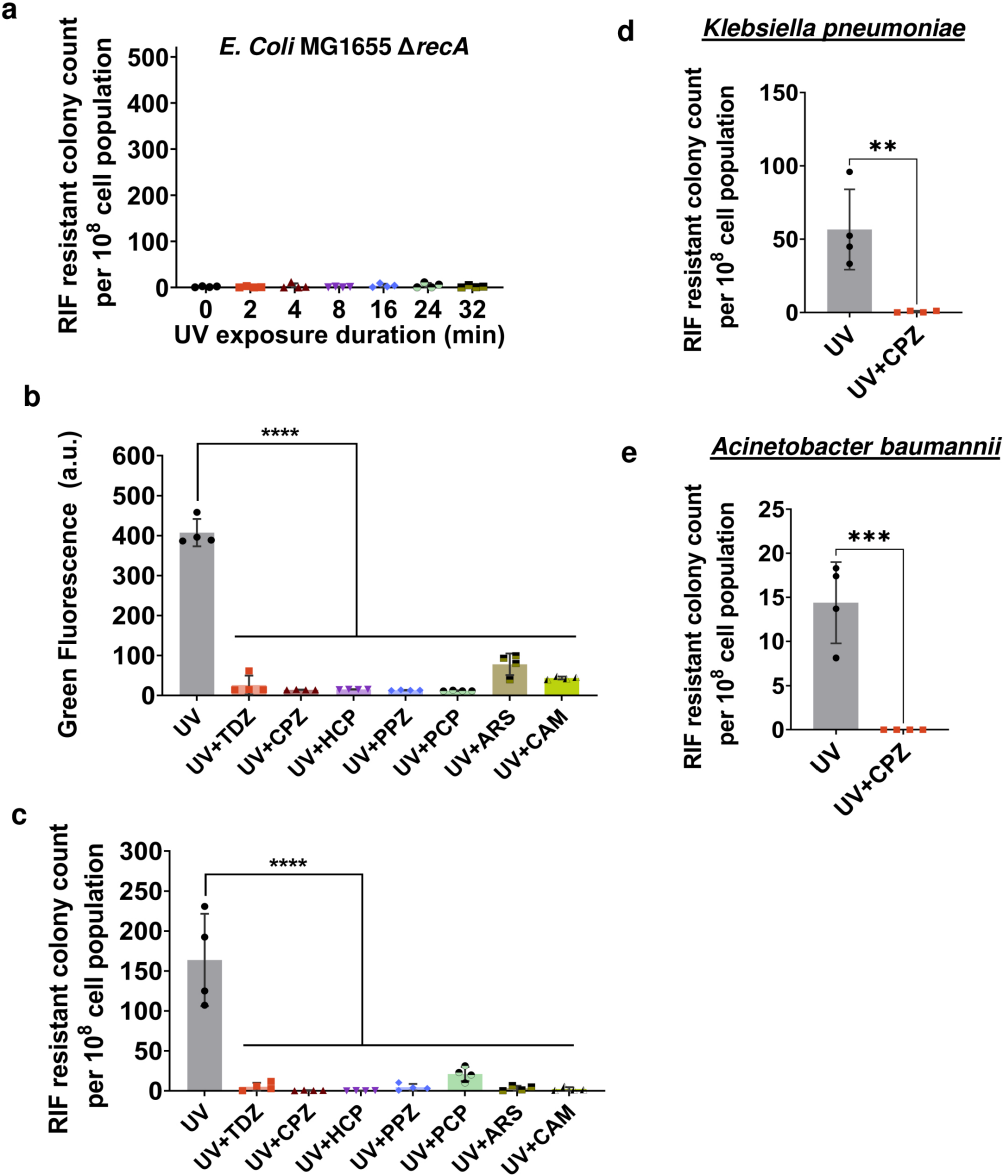
Chemical inhibition of RecA expression can significantly reduce UV-induced SOS mutagenesis. (**a**) Exponential-phase *E. coli* MG1655 Δ*recA* cells were exposed to UV for 0, 2, 4, 8, 16, 24, and 32 min and then, recovered for 24 h. At the end of recovery, cells were plated on RIF agar plates to quantify RIF-resistant colonies. (**b**) Mid-exponential phase *E. coli* MG1655 pUA66- P*_recA_-gfp* cells were treated with chemical inhibitors namely, thioridazine (TDZ; 1 mM), chlorpromazine (CPZ; 0.25 mM), hexachlorophene (HCP; 0.1 mM), perphenazine (PPZ; 1 mM), pentachlorophenol (PCP; 0.3 mM), arsenate (ARS; 1 mM), and chloramphenicol (CAM; 20 µg/mL) and exposed to UV radiation for 16 min and recovered for 24 h. After 24 h, *recA* expression levels were measured with a plate reader. (**c**) Quantification of mutant cells involved plating washed samples onto RIF agar plates after a 24-h recovery period. (**d and e**) Mid-exponential phase cells of *Klebsiella pneumoniae* (CXY 130 WT) and *Acinetobacter baumannii* (BAA-1605 WT) were treated with CPZ (0.3 mM and 0.1 mM, respectively) and UV for 16 min and then recovered for 24 h. RIF-resistant colonies were quantified in each case by plating the washed samples after 24 h of recovery onto RIF agar plates. *n* = 4. Pairwise statistical significance was performed using one-way ANOVA with Dunnett’s post-test. **, *P*  <  0.01; ***, *P*  <  0.001; ****, *P*  <  0.0001. Data corresponding to each column represent mean value  ± standard deviation.

The identified correlation between SOS response and mutagenesis presents a potential strategy to minimize the formation of mutant cells. UV-induced mutation requires functional UmuC and UmuD, forming a DNA polymerase specialized for trans-lesion synthesis in *E. coli* (20, 21). RecA is essential for the UmuC/UmuD-mediated repair mechanism (13, 14). As anticipated, the knockout strains of *E. coli* BW25113, specifically Δ*umuC*, Δ*umuD*, and Δ*recA* (obtained from the Keio Collection), demonstrated a notable reduction in mutagenesis upon UV exposure compared to both the wild type (WT) and the Δ*sulA* strain (Fig. S5). The Δ*sulA* strain was used as a control, as SulA is not directly involved in DNA repair. Given that RecA serves as the global regulator of the SOS response in *E. coli*, deleting *recA* completely eradicated mutagenesis in the *E. coli* MG1655 strain (Fig. 2a), similar to the findings in the BW25113 strain (Fig. S5). During the recovery of cells, transcription and translation should become crucial for the expression of DNA repair genes. By employing bacteriostatic inhibitors like chloramphenicol (CAM) to inhibit transcription/translation processes (39, 40), mutagenesis can be effectively eliminated. Furthermore, depletion of ATP using chemicals like arsenate (ARS) (41, 42) may impede repair as RecA depends on ATP for catalyzing the DNA strand-exchange reaction. Phenothiazine drugs like thioridazine (TDZ), chlorpromazine (CPZ), and perphenazine (PPZ), as well as antiseptic chemicals like hexachlorophene (HCP) and pentachlorophenol (PCP), were shown to indirectly inhibit *recA* expression in fluoroquinolone-treated *E. coli* by impacting energy metabolism and the SOS response (43). To assess the effects of these metabolic inhibitors on mutagenesis, mid-exponential *E. coli* MG1655 cells carrying the P*_recA_-gfp* reporter plasmid were treated with 1 mM TDZ, 0.25 mM CPZ, 0.1 mM HCP, 1 mM PPZ, 1 mM ARS, 0.3 mM PCP, and 20 µg/mL CAM and immediately exposed to 16 min of UV radiation. A cell culture exposed to UV without any chemical treatment served as the control group. The indicated non-lethal concentrations of the chemicals were chosen to optimize their impact on transcription/translation and energy metabolism in the cells (43). Following UV treatment, the cells were subjected to a 24-h recovery period in the presence of chemicals, after which they were washed to remove the chemicals and plated on RIF agar plates. TDZ, CPZ, HCP, and PPZ treatments exhibited no significant impact on cell viability when compared to the control group that underwent recovery without any chemicals following UV treatment (Fig. S6). Conversely, ARS, PCP, and CAM treatments showed a 10-fold difference in CFU levels compared to the control, likely attributable to the bacteriostatic effects of these drugs (Fig. S6). Therefore, to maintain comparable CFU levels with the ARS-, PCP-, and CAM-treated cultures, the control was diluted 10-fold before transfer to RIF plates. Our results demonstrated a significant reduction in *recA* promoter activity during recovery for all chemical treatments, consistent with our expectations (Fig. 2b). These metabolic inhibitors also effectively reduced UV-induced mutations, providing further support for our hypothesis (Fig. 2c).

To evaluate the broader effectiveness of these metabolic inhibitors, we selected two highly pathogenic isolates, namely *Klebsiella pneumoniae* (CXY 130) and *Acinetobacter baumannii* (BAA-1605). For the control group, both strains were exposed to 16 min of UV radiation following a similar protocol, followed by a 24-h recovery period. Co-treatment with CPZ (which was chosen due to its minimal effect on CFU levels during recovery) and 16 min of UV radiation resulted in a 50-fold decrease in RIF-resistant mutant levels in *K. pneumoniae* and a 15-fold decrease in *A. baumannii* (Fig. 2d and e). While we did not investigate all conditions and chemicals for these pathogenic organisms, our results suggest the existence of potential conservation of similar mechanisms across diverse bacterial species.

### The timing of inhibiting *recA* expression is crucial for the efficacy of drugs in reducing mutagenesis

**Fig 3:**
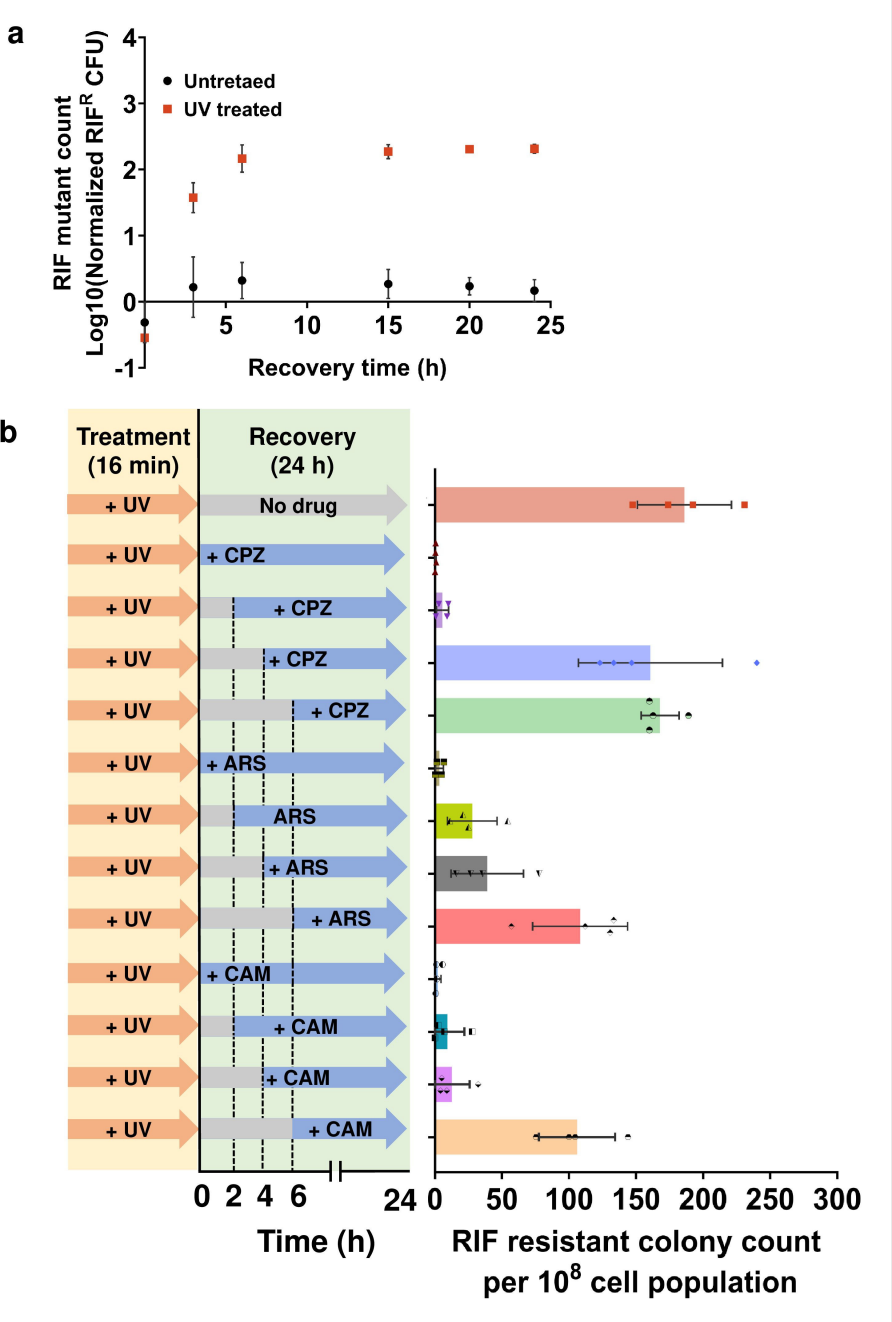
Timing of targeting cellular processes is critical for the effectiveness of the drugs in reducing mutagenesis. (**a**) Mid-exponential phase *E. coli* MG1655 cells were exposed to UV radiation for 16 min and recovered for 24 h. An untreated sample was kept as a control. Samples were taken at indicated time points during recovery and plated on RIF agar medium to quantify the extent of UV-induced mutagenesis. (**b**) Mid-exponential phase *E. coli* MG1655 cells were exposed to UV for 16 min and the metabolic inhibitors, chlorpromazine (CPZ; 0.25 mM), arsenate (ARS; 1 mM), and chloramphenicol (CAM; 20 µg/mL) were added at the indicated time points (as shown in the schematics) during the recovery (0, 2, 4, and 6 h). After 24 h of recovery, mutant cells were quantified by washing the recovered samples and then plating them on the RIF agar medium. *n* = 4. Data corresponding to each column represent mean value  ± standard deviation.

While the inhibition of transcription/translation or depletion of energy molecules at the onset of treatment significantly reduces SOS response activation and mutagenesis, it remains uncertain whether targeting these processes later in the recovery phase yields the same effect. We believe that the timing of targeting these cellular processes is critical for the effectiveness of the drugs in reducing mutagenesis. Early intervention can disrupt mutagenic processes triggered by treatment-induced DNA damage. However, targeting these processes later may be less impactful due to the initiation or completion of DNA repair and mutagenic processes. When quantifying the RIF-resistant colonies formed at specific time points during the recovery of both untreated and 16-min UV-treated cultures, we observed a noticeable increase in RIF-resistant colonies within the 4-h mark in the recovery period (Fig. 3a). Assuming that resistant cells initially exist in a proliferative state with a growth rate similar to wild-type cells, it is conceivable that these cells might appear at earlier time points, and the reported resistant cells at later time points indicate the clonal expansion of these cells with the increasing density of the culture. This suggests that early intervention is likely necessary to disrupt mutagenic processes triggered by treatment. To investigate this further, we conducted an experiment where metabolic inhibitors (0.25 mM CPZ, 1 mM ARS, and 20 µg/mL CAM) were introduced to the 16-min UV-treated cell cultures at different stages of recovery: 0, 2 h, 4 h, and 6 h (Fig. 3b). Following a 24-h recovery period, the cultures were plated to quantify both the total number of cells (refer to Fig. S7) and RIF-resistant colonies (Fig. 3b), as detailed in previous sections. The addition of inhibitors at earlier time points (0 and 2 h) resulted in a significant elimination of UV-induced mutagenesis as predicted, whereas introducing the inhibitors after 4 h and 6 h of recovery showed a relatively higher level of RIF-resistant mutants (Fig. 3b), supporting our claims.

### Fluoroquinolone antibiotics induce enhanced mutagenesis compared to conventional antibiotics but result in fewer mutational events compared to UV treatment

**Fig 4:**
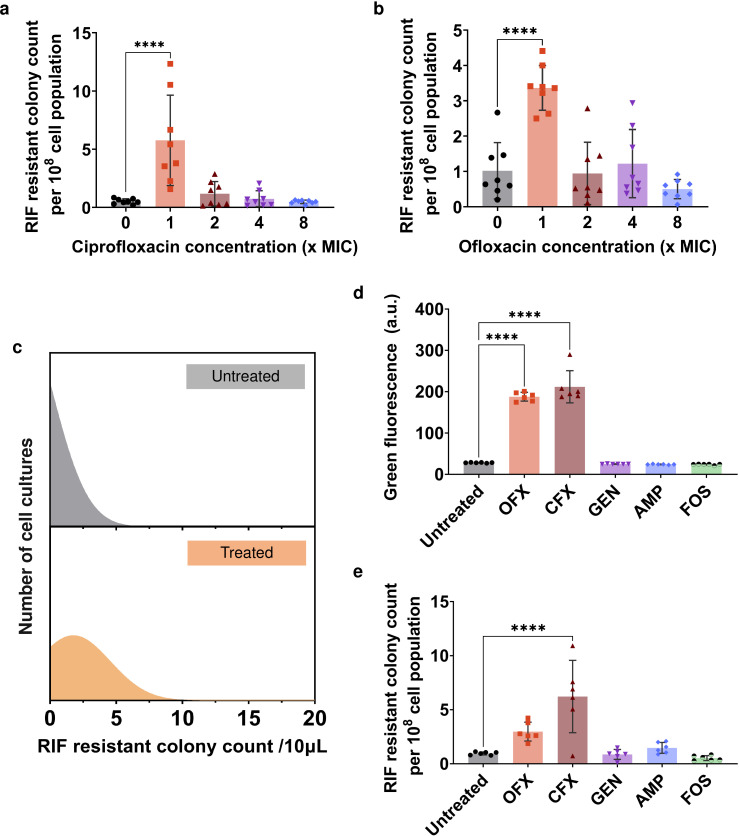
Fluoroquinolone antibiotics induce enhanced mutagenesis compared to conventional antibiotics. (**a and b**) *E. coli* MG1655 cells were treated with ciprofloxacin (CFX; MIC: 0.02 µg/mL) and ofloxacin (OFX; MIC: 0.07 µg/mL) at indicated concentrations for 24 h, then washed and recovered for 24 h in fresh media. Antibiotic-induced SOS mutagenesis was quantified by plating recovered cells on RIF agar plates. (**c**) Validation of the correlation between the CFX-induced SOS response and mutagenesis levels utilizing a 96-well plate format. A total of 384 separate cell cultures underwent treatment with 1× MIC of CFX for 24 h, followed by a 24-h recovery in antibiotic-free fresh media. A parallel process was applied to the untreated control, using solvent instead of CFX. After the recovery period, a 10 µL sample from each culture was plated onto RIF agar plates. A frequency distribution was generated from the RIF-resistant colony data for the two experimental conditions and non-linear regression using a Gaussian distribution was conducted to obtain the probability distribution. (**d and e**) *E. coli* MG1655 pUA66- *P_recA_-gfp* cells were treated with 1× MIC of conventional antibiotics, ciprofloxacin (CFX; MIC: 0.02 µg/mL), ofloxacin (OFX; MIC: 0.07 µg/mL), gentamycin (GEN; MIC: 0.5 µg/mL), ampicillin (AMP; MIC: 6 µg/mL), and fosfomycin (FOS; MIC: 0.1 µg/mL), for 24 h, and, then washed and recovered for 24 h in fresh media. After 24 h of recovery, *recA* expression levels were measured and antibiotic-induced SOS mutagenesis was quantified by plating recovered cells on RIF agar plates. *n* = 6, Pairwise statistical significance was performed using one-way ANOVA with Dunnett’s post-test. ****, *P*  <  0.0001. Data corresponding to each time point represent mean value  ± standard deviation.

Next, we investigated intracellular mutagenesis facilitated by conventional antibiotics. Our initial focus was on fluoroquinolone antibiotics, such as ofloxacin and ciprofloxacin, which act directly on bacterial DNA gyrase, causing DNA damage and promoting mutagenesis. To ensure significant exposure to antibiotic-induced damage, we treated *E. coli* cells with increasing concentrations of fluoroquinolones, ranging from 1× Minimum inhibitory concentration (MIC) up to 8× MIC, for a duration of 24 h. We opted for this extended treatment period to enhance the mutagenesis rates. Following the treatment, we carefully washed the cells to remove any remaining drugs and then resuspended them in fresh media, allowing for an additional 24 h of recovery. Recovery also proves to be crucial, as we observed an absence of RIF-resistant mutants when cells were directly plated on the RIF agar medium immediately after treatment. Our findings revealed that for both ciprofloxacin and ofloxacin, the highest number of RIF-resistant mutants emerged at 1× MIC concentration (Fig. 4a and b), although the reduced number of bacterial cells surviving at higher antibiotic concentrations may decrease the probability of mutational events (Fig. S8a and b). However, this outcome is still in line with the dose-dependent mutagenesis observed in both UV and H_2_O_2_ treatments. To further confirm the increased mutagenesis at 1× MIC, we determined the frequency distribution of mutagenesis using the method based on the 96-plate format, as previously described. Given the similar trends observed in both ofloxacin and ciprofloxacin for the induction of intracellular mutagenesis, we chose to focus on ciprofloxacin here. Our frequency distribution plot showed a clear difference between the untreated samples and those treated with ciprofloxacin, while the treated samples exhibited a much wider distribution (Fig. 4C; Fig. S9). Although ciprofloxacin demonstrated the highest level of RIF-resistant cells at 1× MIC, it still results in fewer mutational events compared to UV radiation (Fig. 1d; Fig. S3).

We also wanted to compare the SOS response and mutation rates induced by fluoroquinolones to those of other conventional antibiotics, such as gentamicin (an aminoglycoside that inhibits protein biosynthesis by binding to the 30S ribosomal subunit), ampicillin (a β-lactam that inhibits cell wall biosynthesis by binding to penicillin-binding proteins), and fosfomycin (a phosphonic acid that blocks cell wall biosynthesis by inhibiting the initial step involving phosphoenolpyruvate synthetase). Following a 24-h treatment with antibiotics at 1× MIC and a subsequent 24-h recovery period, cell viability remained consistent across all conditions, comparable to the untreated culture (Fig. S10). However, we observed a significant difference in *recA* expressions between these antibiotics and fluoroquinolones in the recovery cultures (Fig. 4d). Specifically, fluoroquinolones exhibited a significant upregulation in *recA* expressions, showing an approximately 7.5-fold and 6.5-fold increase compared to the untreated control. By contrast, treatment with gentamicin, ampicillin, and fosfomycin did not result in a similar upregulation in *recA* expression. This disparity in *recA* expression values aligns with the observations in RIF-resistant mutants, where only cells treated with ciprofloxacin and ofloxacin showed a 6.5-fold and 3-fold increase in resistance levels, respectively, compared to the untreated control (Fig. 4e). These results are consistent with the expected outcomes based on the *recA* expression values of fluoroquinolone-treated cells.

### Metabolic inhibitors can also reduce antibiotic-induced mutagenesis

**Fig 5:**
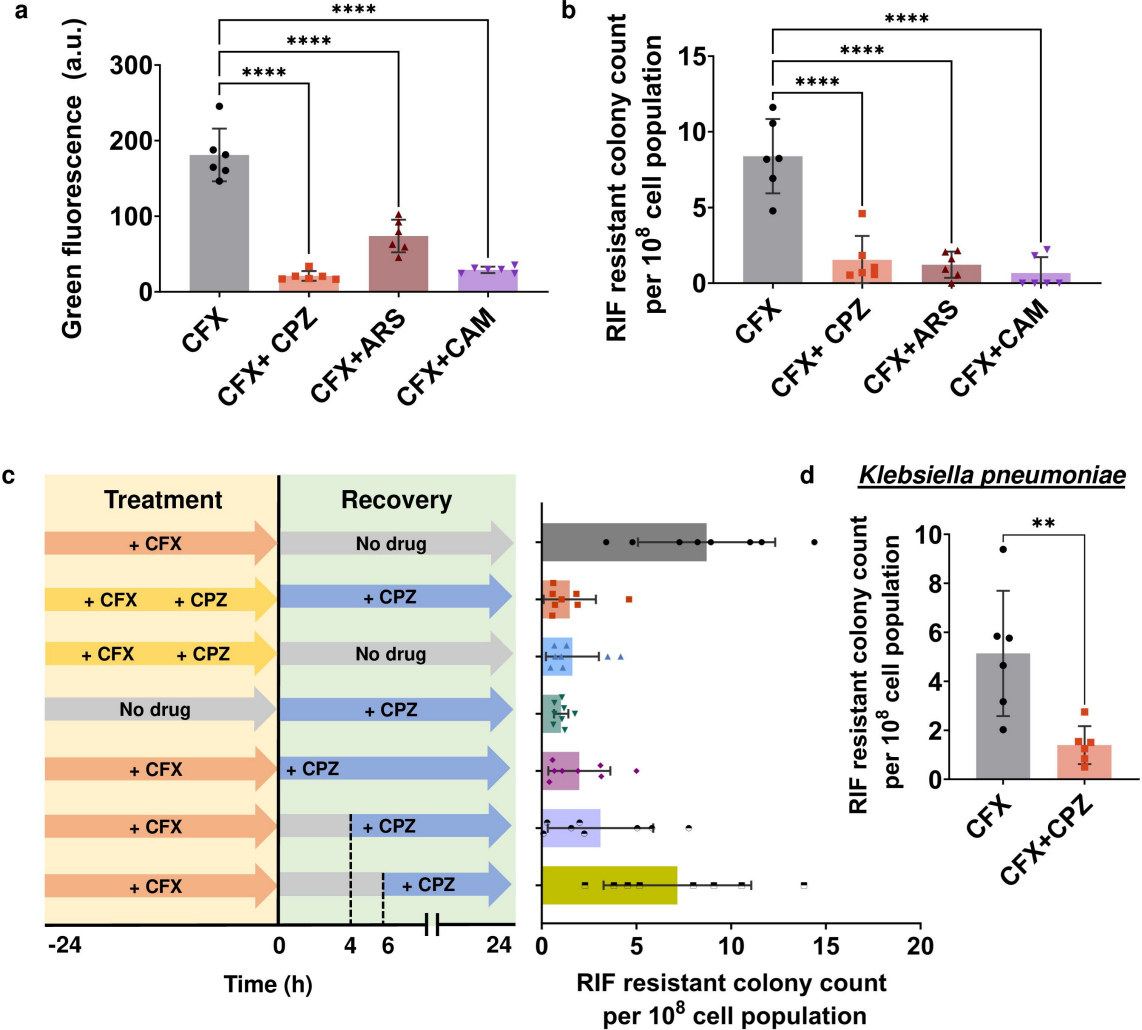
Co-treatment with metabolic inhibitors reduced *recA* expression levels and antibiotic-induced mutagenesis. (**a and b**) *E. coli* MG1655 pUA66- *P_recA_-gfp* cells were co-treated with 1× MIC of ciprofloxacin (CFX; 0.02 µg/mL) and metabolic inhibitors, chlorpromazine (CPZ; 0.25 mM), arsenate (ARS; 1 mM), and chloramphenicol (CAM; 20 µg/mL), for 24 h, followed by a 24-h recovery period in fresh media, during which the metabolic inhibitors were also introduced into the recovery cultures. After 24 h of recovery, *recA* expression levels were measured and antibiotic-induced SOS mutagenesis was quantified by plating recovered cells on RIF agar plates. (**c**) *E. coli* MG1655 pUA66- *P_recA_-gfp* cells were co-treated with CFX (1× MIC) and CPZ (0.25 mM) following the experimental schematics shown on the panel. (**d**) Mid-exponential phase cells of *Klebsiella pneumoniae* (CXY 130 WT) were co-treated with 1× MIC of ciprofloxacin (CFX; 3.5 µg/mL) and CPZ (0.3 mM) for 24 h and then recovered for 24 h in fresh media with CPZ. After 24 h of recovery, antibiotic-induced SOS mutagenesis was quantified by plating recovered cells on RIF agar plates. *n* = 6 and 8. Pairwise statistical significance was performed using one-way ANOVA with Dunnett’s post-test and unpaired *t*-test. **, *P*  <  0.01, ****, *P*  <  0.0001. Data corresponding to each time point represent mean value  ± standard deviation.

Finally, we sought to test the effectiveness of the metabolic inhibitors in eliminating ciprofloxacin-induced mutagenesis. We used mid-exponential *E. coli* MG1655 cells containing the P*_recA_-gfp* plasmid and treated them with 0.02 µg/mL ciprofloxacin (equivalent to 1× MIC) in combination with three metabolic inhibitors: 0.25 mM CPZ, 1 mM ARS, and 20 µg/mL CAM. Following the treatment, we allowed the cells to recover for 24 h in the absence of antibiotics while maintaining the inhibitors within the cultures. Compared to the GFP fluorescence level of the control group after the 24-h recovery period, co-treatment of the cells with ciprofloxacin and the metabolic inhibitors led to a significant reduction in *recA* promoter activities (Fig. 5a). Also, these metabolic inhibitors were able to reduce the ciprofloxacin-induced mutations (Fig. 5b). To investigate the impact of inhibitor addition timing on mutagenesis, we introduced CPZ into ciprofloxacin-treated cultures during different recovery stages: 0, 2 h, 4 h, and 6 h. The results indicate that introducing the inhibitor after 4 h and 6 h of recovery led to a relatively higher level of RIF-resistant mutants, consistent with the observed pattern in UV-treated cultures (Fig. 5c; Fig. S11). To further evaluate the broader effectiveness of this metabolic inhibition, we tested them on *K. pneumoniae* (CXY 130). Co-treating *K. pneumoniae* cells with ciprofloxacin at 1× MIC, along with CPZ, resulted in a fourfold reduction in RIF-resistant mutant levels (Fig. 5d). For the *A. baumannii* (BAA-1605) strain, we were unable to apply this method due to its high resistance against fluoroquinolone antibiotics. Altogether, our findings highlight the potential of inhibiting cell metabolism as a strategy to mitigate intracellular mutant formation caused by certain antibiotics.

## DISCUSSION

This study explores the role of the SOS response in DNA damage repair and its connection to antibiotic resistance. We subjected bacterial cells to UV, observing that mutagenesis mainly occurs during the recovery phase. Inhibiting transcription/translation or depleting energy molecules during early recovery reduced mutagenesis, suggesting their timing is critical for effectiveness. These inhibitors also prevented mutagenesis caused by fluoroquinolone antibiotics, indicating the dependence of SOS response and repair mechanisms on active transcription, translation, and energy availability. Our study aligns with a prior publication that emphasizes the significance of timing in events like DNA repair after fluoroquinolone treatment for cell survival and supports the notion that understanding the post-antibiotic recovery period is essential in comprehending tolerance mechanisms (23, 25). Despite some variations in the outcomes of different mutagenic treatments or when using different strains, we observed a consistent correlation between *recA*-mediated SOS response and mutagenesis. Increased expression of *recA* corresponded to higher mutagenesis rates, while the absence of *recA* expression during excessive stress resulted in reduced mutagenesis. This direct correlation holds clinical significance, as manipulating *recA* expression levels could effectively reduce the formation of mutant cells.

We noted a significant decrease in mutagenesis with excessive treatments (such as prolonged UV exposure and higher fluoroquinolone and H_2_O_2_ concentrations), consistent with a previous study (44). Excessive treatments result in a more potent bactericidal effect, leading to a greater reduction in viable bacterial cells, which may limit the pool of potential mutant cells, thereby reducing mutagenesis. In fact, at higher concentrations of mutagenetic factors, the severity of DNA damage may cause cell death before mutations can occur. Moreover, bacterial cells possess DNA repair mechanisms that counteract mutagen-induced damage, and at lower chemical concentrations, cells may have a better chance of surviving DNA damage and initiating mutagenic processes. However, this explanation alone may not account for the observed reduction in mutagenesis during excessive treatments. For instance, despite observing a similar growth deficiency in both 24 mM and 32 mM H_2_O_2_ treatments, we noted a significant number of mutations in the 24 mM treatment compared to the 32 mM treatment. The relationship between growth deficiency and mutagenesis appears more intricate, particularly in the case of UV treatments. Although elevated RecA levels up to the 16-min UV exposure indicate a typical response to DNA damage, our findings reveal that exposure to 24 or 32 min of UV radiation results in impaired cell colonization on agar plates. However, these growth deficiencies are transient, as similar growth patterns were observed after 15 min of recovery across almost all UV treatment conditions. This phenomenon likely provides a survival advantage from an evolutionary perspective, as cells experience reduced damage when entering a dormant state under extreme stress conditions. Multiple factors may contribute to this adaptive response, including the activation of negative feedback loops, and impairment of essential cellular processes due to extensive damage. Indeed, this intriguing outcome can be anticipated, considering the complex nature of the SOS response, which encompasses various genes capable of inducing dormancy, inhibiting cell growth, transcription/translation, and directly or indirectly preventing excessive SOS responses (14). Furthermore, bacterial cultures exhibit high heterogeneity, with a small fraction of the cell population that does not rapidly grow in cultures (45). These cells may not be impacted by longer exposures to UV or high doses of H_2_O_2_ due to their reduced cellular functions. The heterogeneity of bacterial cells, coupled with potential indirect feedback inhibitions and alterations in regulatory proteins, may collectively contribute to the observed non-linear relationship between stress levels and the SOS response, highlighting the need for further investigation in this area.

The findings highlight the potential of inhibiting cell metabolism as a strategy to reduce intracellular mutant formation caused by mutagenic factors, including antibiotics, although the effectiveness of the method may vary depending on the bacterial strains, the timing of treatment, and the inhibitors used. Unfortunately, bacteriostatic chemicals like CAM and ARS are known to induce cell dormancy and promote the formation of antibiotic-tolerant cells, such as persisters, in cultures (41, 42, 46). However, our prior study showed that using phenothiazine drugs in combination with fluoroquinolones can effectively eliminate persister cells (43). Persisters represent a small subpopulation of phenotypic variants capable of surviving antibiotic treatments. While they may be non-growing during antibiotic exposure, these cells can regain the ability to initiate cell growth when transitioning to fresh media and can potentially establish populations of antibiotic-sensitive bacterial cells (47, 48). Previous studies suggest that persister cells experience antibiotic-induced damage similar to antibiotic-sensitive subpopulations, and their survival and colonization rely on DNA repair mechanisms (23, 24). These repair processes involve the reactivation of transcription/translation and the production of energy molecules. Phenothiazine drugs appear to reduce these components to subthreshold levels in antibiotic-damaged persisters, leading to their elimination (43). By contrast, CAM and ARS, across various concentrations, do not effectively eliminate persisters nor reduce cellular ATP levels and transcription/translation activities to the same extent as phenothiazine-treated cultures (43).

Fluoroquinolones yielded a lower number of RIF-resistant colonies compared to UV, suggesting that the drug treatment induced fewer and more limited types of mutations than those caused by UV exposure. This difference is likely due to the distinct mechanisms of action of these two mutagenic agents. UV radiation can cause direct DNA damage, such as the formation of dimers, which can lead to a wide range of mutations, including base substitutions, deletions, and insertions (27, 28). Consequently, this can lead to a diverse array of genetic changes in bacterial cells. On the other hand, the mutagenic effects of drugs, such as fluoroquinolones, may be more targeted and specific. These drugs typically interfere with DNA replication and DNA gyrase, leading to specific types of DNA damage, such as double-stranded DNA breaks (49–51). As a result, the mutations induced by drug treatment may be more limited in variety and scope compared to UV exposure. The variation in mutation spectra between UV exposure and drug treatment underscores the importance of considering the specific mutagenic agent and its mode of action when studying mutagenesis in bacterial cells. Understanding the mechanisms of mutagenesis induced by different agents can provide valuable insights into the development of drug resistance and the overall evolution of bacterial populations.

The significant upregulation of *recA* and higher levels of resistance observed with fluoroquinolones compared to other conventional antibiotics can be attributed to the specific mechanisms of action of fluoroquinolones. Other conventional antibiotics such as gentamicin, ampicillin, and fosfomycin have different modes of action. These antibiotics do not directly induce the same DNA damage and subsequent SOS response seen with fluoroquinolones. As a result, they may not trigger the upregulation of *recA* to the same extent, leading to lower levels of resistance compared to fluoroquinolones. Interestingly, a prior study reported increased mutagenesis induced by ampicillin (52). However, this effect was attributed to the specific culture conditions chosen to maximize aeration and facilitate ROS formation (52).

Our results also reveal that higher concentrations of fluoroquinolones lead to reduced mutagenesis compared to lower concentrations. However, it is noteworthy that mutant strains can still emerge under higher fluoroquinolone concentrations or other antibiotic treatments through various mechanisms. For instance, cyclic exposure to antibiotics is a crucial strategy in laboratory experiments to simulate the evolutionary dynamics of antibiotic resistance, particularly for conventional antibiotics (53–57). This process involves subjecting bacterial populations to intermittent or repeated rounds of antibiotic treatment, followed by recovery periods without antibiotics. This mimics real-world scenarios where bacterial infections are exposed to antibiotic treatment cycles due to intermittent dosing or patient compliance. Over time, the repeated cycles of exposure and recovery create an environment that favors the survival and proliferation of resistant mutants. These mutants may carry genetic changes that alter the antibiotic target site, enhance antibiotic efflux, or modify cell wall composition, making them less susceptible to the antibiotic’s effects. Consequently, the population becomes enriched with resistant strains, leading to reduced effectiveness of the antibiotic over successive treatment cycles.

In conclusion, this study shows that simple strategies, like early inhibition of transcription/ translation and energy depletion, effectively reduce mutagenesis, emphasizing their vital role in SOS response activation and repair mechanisms. However, as with any chemical intervention, there is a risk of bacteria developing resistance mechanisms through mutations. While our research sheds light on treatment strategies such as the timing of the SOS response inhibition, it also highlights the promising avenue of exploring diverse mechanisms for potential drug development.

## MATERIALS AND METHODS

### Bacterial strains and plasmids

*Escherichia coli* K-12 MG1655 WT and pUA66 plasmids with genes encoding a green fluorescent protein (*gfp*) under the control of three promoters, P*_recA_*, P*_sulA_*, and P*_tisB_*, were obtained from Dr. Mark P. Brynildsen at Princeton University. *E. coli* K-12 MG1655 Δ*recA* constructed in our previous studies (43, 58). *Klebsiella pneumoniae* (CXY 130) and *Acinetobacter baumannii* (BAA-1605) isolates were obtained from Dr. Kevin W. Garey at the University of Houston. *E. coli* K12 BW25113 WT, Δ*recA*, Δ*umuC*, Δ*umuD*, and Δ*sulA* strains were acquired from the Keio Knockout Collection (59). The bacterial strains used in this study are detailed in Table S1.

### Chemicals, media, and culture conditions

Unless otherwise specified, all chemicals were procured from Fisher Scientific (Atlanta, GA), VWR International (Pittsburgh, PA), or Sigma Aldrich (St. Louis, MO). *E. coli* cells were cultured in liquid LB medium, while *K. pneumoniae* and *A. baumannii* cells were cultivated in liquid Mueller-Hinton (MH) medium. LB agar medium was utilized for enumerating CFU of *E. coli*, whereas MH agar medium was employed for *K. pneumoniae* and *A. baumannii* cells. The LB broth was prepared by dissolving 5 g yeast extract, 10 g tryptone, and 10 g sodium chloride in 1 L of deionized (DI) water. The MH broth was prepared by dissolving 2 g beef extract powder, 17.5 g acid digest of casein, and 1.5 g soluble starch in 1 L of DI water. Agar media were prepared by dissolving pre-mixed 40 g LB agar or 38 g MH agar in 1 L of DI water, respectively. Both solid and liquid media were subjected to autoclaving for sterilization.

Kanamycin (50 µg/mL) was included in the liquid LB media for plasmid selection and retention. When necessary, cells were washed with phosphate-buffered saline (PBS, 1×). A 3% wt/vol H_2_O_2_ stock solution was used to prepare H_2_O_2_ concentrations of 15 mM, 30 mM, 60 mM, 120 mM, 240 mM, and 320 mM in LB medium. Stock solutions for ampicillin (60 mg/mL), fosfomycin (10 mg/mL), and gentamycin (50 mg/mL) were prepared in DI water. Ofloxacin (5 mg/mL), ciprofloxacin (20 mg/mL), and rifampicin (RIF; 50  mg/mL) were dissolved in DI water using 0.01 N sodium hydroxide. Thioridazine (TDZ; 0.1 M), chlorpromazine (CPZ; 0.5 M), and arsenate (sodium hydrogen arsenate heptahydrate, 1 M) were prepared in DI water. Chloramphenicol (CAM; 50  mg/mL) was dissolved in ethanol (100%). Perphenazine (PPZ; 0.5 M), hexachlorophene (HCP; 0.1 M), and pentachlorophenol (PCP; 0.5 M) stock solutions were made in dimethyl sulfoxide (DMSO). All chemical solutions were sterilized using 0.2 µm VWR syringe filters, except for those dissolved in DMSO. MICs of antibiotics for different strains are provided in Table S2. The twofold macro-dilution method as described before was used to determine the MIC levels (60).

To prepare RIF agar plates, the stock RIF solution was added to autoclaved LB agar and MH agar, resulting in a final plate concentration of 500 µg/mL RIF. Unless specified otherwise, overnight pre-cultures were generated in 14 mL Falcon test tubes containing 2 mL of liquid media. These pre-cultures were inoculated from 25% glycerol cell stocks stored at −80°C and cultivated for 24  h at 37°C with shaking at 250 revolutions per min (rpm). Experimental cell cultures were prepared by diluting the overnight pre-cultures (1:100) into 2 mL of fresh LB medium in 14 mL test tubes. Bacterial cells in this study reached the mid-exponential phase (OD_600_ ~0.5) after around 3 h, attaining an average cell density of 7 × 10^8^ CFUs/mL. All treatments involving UV, H_2_O_2_, and antibiotics (see below) were administered at this stage.

### UV treatment and cell recovery

Overnight pre-cultures of *E. coli* MG1655 cells were diluted 100-fold in 2 mL fresh LB media in test tubes and grown at 37°C with shaking (250 rpm). Cell growth was monitored by measuring optical density at 600 nm wavelength (OD_600_) with a plate reader (Varioskan LUX Multimode Microplate Reader, Thermo Fisher, Waltham, MA, United States). When the cell density reached OD_600_ ~ 0.5, cultures from the test tubes were transferred to Petri dishes (the diameter of Petri dishes = 100 mm, catalog no. FB0875713, Fisher Scientific), creating a thin film of culture in the dish with a height of about 0.25 mm. Then, these cultures were exposed to UV light (UVP ChemStudio, catalog no. 849–97-0928-02; Analytik Jena, Jena, Germany) for varying exposure times (0, 2, 4, 8, 16, 24, and 32 min). After UV exposure, cells were transferred back to test tubes and recovered for 24 h. During the recovery period, 10 µL samples were collected at specified time points from each test tube, serially diluted in PBS in round-bottom 96-well plates, and then, plated on LB agar media. The plates were incubated for at 37°C for 16 h to enumerate CFU. We note that new colonies were not formed when incubated beyond 16 h. A similar procedure was followed for the other strains.

### Fluorescent protein expression assay for SOS reporters

Overnight pre-cultures of *E. coli* MG1655 cells containing *gfp* genes fused to SOS promoters (P*_recA_*, P*_sulA_*, and P*_tisB_*) were diluted 1:100 in 2 mL of LB media within test tubes and incubated at 37°C with shaking (250 rpm). Upon reaching the mid-exponential phase, the cells were subjected to UV radiation or treated with specified drug concentrations when indicated. During the recovery period, 200 µL of cell cultures was sampled at predetermined time intervals and transferred to a flat-bottom 96-well plate. The measurement of GFP expression was carried out using a plate reader (Varioskan LUX Multimode Microplate Reader) with excitation and emission wavelengths set at 485 nm and 511 nm, respectively. Data from the plate reader were recorded using SkanIt Software V 5.0. Whenever specified, GFP expression was normalized by dividing it by the cell density (OD_600_) at the specified time during recovery.

### Mutant quantification

To assess the extent of mutant cell formation induced by UV exposure or antibiotic treatment, cells were exposed to UV radiation or subjected to chemical mutagens for specified durations, followed by a 24-h recovery period. After this recovery period, 500 µL of cells was collected and washed three times to eliminate any residual chemicals. Subsequently, the cells were spread onto agar plates containing 500 µg/mL of RIF. These plates were then incubated at 37°C for 16 h to enumerate RIF-resistant colonies. To determine clonogenic survival, CFU levels were determined, and mutant levels were normalized by dividing the number of RIF-resistant colonies by the total number of colonies in a 1 mL culture volume. Unless specified otherwise, mutant formation was reported as the count of RIF-resistant colonies per 10^8^ cell population.

### UV treatment methodology for 96-well plates

Overnight pre-cultures of *E. coli* MG1655 pUA66-P*_recA_-gfp* cells were diluted 40-fold into flat-bottom 96-well plates, with each well containing 200 µL of cell culture. The plates were securely sealed using a sterile, oxygen-permeable membrane (Breathe-Easier, Cat# BERM-2000, VWR International) and incubated at 37°C with agitation at 250  rpm. When the cultures reached the mid-exponential phase (OD_600_ ~0.5), the cells in the 96-well plates were exposed to UV light (UVP ChemStudio, catalog no. 849–97-0928-02; Analytik Jena, Jena, Germany) for various durations (0, 2, 4, 8, and 16 min). Subsequently, they were allowed to recover for 24 h at 37°C with shaking at 250  rpm. To quantify UV-induced mutant cells, 10 µL of samples was collected from each well and spotted onto LB agar plates containing 500 µg/mL RIF at the end of the 24-h recovery period. The plates were then incubated at 37°C for 16 h to enumerate RIF-resistant colonies. The mutant formation was reported as the count of RIF-resistant colonies per 10 µL of cell culture. This experiment was repeated three times, resulting in a total of 288 independent UV-treated cell cultures.

### UV treatment with varying recovery times

Overnight pre-cultures of *E. coli* MG1655 pUA66-P*_recA_-gfp* cells were diluted 100-fold in 2 mL fresh LB media in test tubes and grown at 37°C with shaking (250 rpm). Cell growth was monitored by measuring OD_600_ with a plate reader (Varioskan LUX Multimode Microplate Reader, Thermo Fisher, Waltham, MA, United States). Mid-exponential phase cells (OD_600_ ~0.5) were then exposed to UV light for 16 min. A culture without UV treatment was kept as a control. During the 24-h recovery, 500 µL of cell suspension was taken out at different time points: 0 h, 3 h, 6 h, 15 h, 20 h, and 24 h (one test tube per time point), and then spread on agar plates with 500 µg/mL RIF. The plates were incubated at 37°C for 16 h to enumerate the RIF-resistant CFU.

### Cotreatment with UV and metabolic inhibitors

Overnight pre-cultures of *E. coli* MG1655 pUA66-P*_recA_-gfp* cells were diluted 100-fold in 2 mL of fresh LB media within test tubes and cultured at 37°C with shaking (250 rpm). When the cell density reached an OD_600_ of 0.5, seven drugs (thioridazine, chlorpromazine, hexachlorophene, perphenazine, pentachlorophenol, arsenate, and chloramphenicol) were added to seven different test tubes at specified concentrations. In the control, only the solvents were added. The drug-treated cultures, along with the control, were then transferred to Petri dishes and exposed to UV light for 16 min. After UV exposure, the cells were transferred back to the test tubes and allowed to recover at 37°C with shaking (250 rpm). In specific cases, CPZ, ARS, and CAM were added after 2 h, 4 h, and 6 h from the start of the recovery culture to investigate the importance of recovery time in the formation of RIF-resistant mutants. Following a 24-h recovery period, 200 µL of cell cultures was transferred to a flat-bottom 96-well plate, and green fluorescence was measured with a plate reader to assess *recA* expression levels at the end of the recovery. Additionally, a 1 mL sample from each Falcon tube was transferred to a microcentrifuge tube and centrifuged at 13,300 rpm. The 950 µL supernatant was removed, and the cell pellet was washed three times with 950 µL PBS to reduce the antibiotic concentration to below MIC. After washing, the cells were resuspended in 1 mL of PBS. A 10 µL sample of this cell suspension was serially diluted in 90 µL PBS in a round-bottom 96-well plate, and then 10 µL of the diluted cells were spotted onto an LB agar plate to count CFU. Cell levels were reported as the number of CFU per 1 mL of the assay culture. The LB agar plates were incubated for at least 16 h at 37°C, as this incubation period was found to be sufficient for the growth of all viable *E. coli* cells. To quantify mutant cells, 500 µL of washed cell suspensions was plated on LB agar plates containing 500 µg/mL RIF and incubated at 37°C for 16 h to enumerate RIF-resistant colonies. Mutant levels were reported as the number of RIF-resistant colonies per 10^8^ cell population.

### Antibiotic treatment and cell recovery

Overnight pre-cultures of *E. coli* MG1655 cells were diluted 100-fold in 2 mL fresh LB media in test tubes and grown at 37°C with shaking (250 rpm). Cell growth was monitored by measuring OD_600_ with a plate reader. When the cell density reached OD_600_ ~ 0.5, cultures were treated with specified antibiotics such as ciprofloxacin, ofloxacin, ampicillin, gentamycin, and fosfomycin at indicated concentrations for 24 h. Only solvents were added to the control. After 24-h treatment, 200 µL of cell cultures was transferred to a flat-bottom 96-well plate and green fluorescence was measured with a plate reader to report the *recA* expression level. Also, the remaining culture from each falcon tube was transferred to a microcentrifuge tube and centrifuged at 13,300 rpm. The supernatant was removed, and the cell pellet was washed three times with PBS to reduce the antibiotic concentration to less than the MIC. After washing, the cells were resuspended in 2 mL fresh LB media and recovered for 24 h. At the end of recovery, 200 µL of cell cultures was transferred to a flat-bottom 96-well plate and green fluorescence was measured with a plate reader to report the *recA* expression level. A 10 µL sample of the cell suspension was serially diluted in 90 µL PBS in a round-bottom 96-well plate and then 10 µL of the diluted cells were spotted on an LB agar plate to count the CFU. Cell levels were reported as the number of CFU per 1 mL of assay culture. The LB agar plates were incubated for at least 16  h at 37 °C, as we found this incubation period was sufficient for the growth of all viable *E. coli* cells. To quantify the mutant cells, 500 µL of washed cell suspensions was plated in LB agar with 500 µg/mL RIF and incubated at 37°C for 16 h to enumerate RIF-resistant colonies. Mutant levels were reported as RIF-resistant colony count per 10^8^ cell population.

### Cotreatment with conventional antibiotics and metabolic inhibitors

A similar pretreatment methodology was followed as mentioned above for the antibiotic treatment. Mid-exponential phase cells were treated with 0.02 µg/mL ciprofloxacin (1× MIC) along with metabolic inhibitors (wherever mentioned) such as CPZ (0.25 mM), ARS (1 mM), and CAM (20 µg/mL) for 24 h. Cell cultures were then washed three times with PBS to reduce the concentrations of the drugs below MIC. Recovery cultures were started by resuspending the washed cell suspensions in 2 mL fresh LB media and the metabolic inhibitors CPZ (0.25 mM), ARS (1 mM), and CAM (20 µg/mL) were added again unless mentioned otherwise. For some specific cases (Fig. 5C), CPZ was added after 4 h and 6 h of the starting of the recovery culture to investigate the importance of recovery time in the formation of RIF-resistant mutants. After 24 h of recovery, the cell cultures were washed again to reduce the concentrations of drugs below MIC. A 10 µL sample of the cell suspensions was serially diluted in 90 µL PBS in a round bottom 96-well plate and then 10 µL of the diluted cells were spotted on an LB agar plate to count CFU. Cell levels were reported as the number of CFU per 1 mL of assay culture. The LB agar plates were incubated at least 16  h at 37°C, as we found this incubation period was sufficient for the growth of all viable *E. coli* cells. To quantify the mutant cells, 500 µL of washed cell suspensions was plated in LB agar with 500 µg/mL RIF and incubated at 37°C for 16 h to enumerate RIF-resistant colonies. Mutant levels were reported as RIF-resistant colony count per 10^8^ cell population.

### Ciprofloxacin treatment methodology for 96-well plates

Overnight pre-cultures of *E. coli* MG1655 pUA66-P*recA-gfp* cells were diluted 40-fold in the flat bottom 96-well plates, with each well containing 200 µL cell culture. Plates were sealed with a sterile, oxygen-permeable membrane (Breathe-Easier, Cat# BERM-2000, VWR International) and incubated at 37°C with shaking at 250 rpm. At the mid-exponential phase (OD_600_ ~0.5), cultures in 96-well plates were treated with 1× MIC of ciprofloxacin (0.02 µg/mL) for 24 h. After treatment, cells in the wells were washed two times in 96-well plates to reduce the ciprofloxacin concentration well below MIC. For the washing steps, the plates were centrifuged at 4,700 rpm for 10 min, and the supernatants from the wells were carefully removed without disturbing the pellets at the bottom, using a multichannel pipette. Subsequently, the cells were resuspended in PBS, and this procedure was repeated. Upon completion of the washing process, the cells were resuspended in fresh LB media and recovered for 24 h at 37°C with shaking at 250 rpm. To quantify the ciprofloxacin-induced mutant cells after recovery, a 10 µL sample was collected from each well and spotted on LB agar with 500 µg/mL RIF at the end of 24-h recovery. The plates were incubated at 37°C for 16 h to enumerate RIF-resistant colonies. A frequency distribution was generated from the RIF-resistant colony data for the two experimental conditions.

### *Klebsiella pneumoniae* and *Acinetobacter baumannii* mutagenesis assay

Overnight cultures of *Klebsiella pneumoniae* (CXY 130) and *Acinetobacter baumannii* (BAA-1605) strains were diluted 1:100 in 2 mL MH in 14 mL Falcon tubes and grown at 37 °C with shaking (250 rpm). For the UV-induced mutagenesis assay, mid-exponential phase (OD_600_ ~0.5) cells were treated with CPZ (0.3 mM and 0.1 mM, respectively) and UV for 16 min and then recovered for 24 h. After the recovery, 1 mL of culture from each tube was collected and washed, serially diluted, and plated on MH agar as described above. Mutant cell formation was quantified in each case by plating the washed samples on a RIF-MH agar plate. The plates were incubated for 20 h at 37°C.

For the CFX-induced mutagenesis assay, mid-exponential phase wild-type cells of *Klebsiella pneumoniae* (CXY 130 WT) were co-treated with 1× MIC of CFX (3.5 µg/mL) and CPZ (0.3 mM) for 24 h and then recovered for 24 h in fresh MH media with CPZ. After 24 h of recovery, 1 mL of culture from each tube was collected and washed, serially diluted, and plated on MH agar. Antibiotic-induced SOS mutagenesis was quantified by plating recovered cells on a RIF-MH agar plate and reported as RIF-resistant colony count per 10^8^ cell population.

### H_2_O_2_ treatment

Overnight pre-cultures of *E. coli* MG1655 pUA66-P*recA-gfp* cells were diluted 100-fold in 1.8 mL fresh LB media in test tubes and grown at 37°C with shaking (250 rpm). Cell growth was monitored by measuring OD_600_ with a plate reader. When the cell density reached OD_600_ ~0.5, cultures were treated with 200 µL of the H_2_O_2_ solutions to make the concentrations 32 mM, 24 mM, 12 mM, 6 mM, 3 mM, and 1.5 mM. For the untreated control, only 200 µL of LB media was added. The cells were washed after 30 min, and, then, recovered for 24 h in fresh media. During the recovery period, 10 µL of samples was collected at specified time points from each test tube, serially diluted in PBS using round-bottom 96-well plates, and then plated on LB agar media. To quantify the mutant cell formation, 250 µL of samples were collected from each tube and spread on LB agar with 500 µg/mL RIF. The plates were incubated at 37°C for 16 h to enumerate CFU. Whenever specified, GFP expression was normalized by dividing it by the cell density (OD_600_) at the specified time during recovery.

### Statistical analysis and reproducibility

For all pairwise comparisons, one-way ANOVA with Dunnett’s post-test was utilized. A minimum of four independent biological replicates (unless otherwise specified) were conducted for experiments involving UV exposure, while a minimum of six biological replicates were performed for experiments involving antibiotic treatments. In all figures (excluding frequency distribution plots), data corresponding to each time point represent the mean value ± standard deviation. For finding the probability distribution of RIF-resistant colony generation across various durations of UV exposure or for ciprofloxacin treatment, a frequency distribution was generated using the RIF-resistant colony data for each experimental condition, and non-linear regression was performed using a Gaussian distribution to obtain the probability distribution of RIF-resistant colony generation for different UV exposure durations. The Gaussian distribution function is $Y=Amplitude*e^{-0.5{\left(\frac{X-Mean}{SD}\right)}^{2}}$ , where, Y = count of RIF-resistant colonies per 10 µL of cell culture, X = number of independent cell cultures, the amplitude is the height of the center of the distribution in Y units, mean is the X value at the center of the distribution, SD or standard deviation is a measure of the width of the distribution, in the same units as X. For experiments involving antibiotics to induce mutagenesis, robust regression, and outlier removal (ROUT) tests were conducted for each case, with a ROUT coefficient Q = 1 (61). This step is crucial because, even in untreated cultures, random mutations can occur. These mutations may result in unusually high levels of RIF-resistant colonies as the progeny of mutant cells proliferate over the extended culture periods (24-h treatment periods followed by 24-h recovery periods) designed for antibiotic treatment experiments. While these instances are very rare, an outlier test is necessary to detect and address such cases. Regarding statistical significance analysis, the threshold values were set as follows: *P  <  0.05, **P  <  0.01, ***P  <  0.001, and ****P  <  0.0001. All figures were created using GraphPad Prism 10.0.2, and the statistical analyses were carried out using GraphPad Prism 10.0.2 statistical functions.

## SUPPLEMENTAL MATERIAL

Supplemental material aac.01462-23-s0001.docx Fig. S1 to S11; Tables S1 and S2.

ASM does not own the copyrights to Supplemental Material that may be linked to, or accessed through, an article. The authors have granted ASM a non-exclusive, world-wide license to publish the Supplemental Material files. Please contact the corresponding author directly for reuse.
