# Supplementary material for: Exploring the links between SOS response, mutagenesis, and resistance during the recovery period
Source: Antimicrob Agents Chemother. 2024 Mar 27;68(5):e01462-23. doi: 10.1128/aac.01462-23 (PMC11064565; doi:10.1128/aac.01462-23)
Supplement: Supplemental material — Fig. S1 to S11; Tables S1 and S2. [file aac.01462-23-s0001.docx]

**Supplementary Information**

**EXPLORING THE LINKS BETWEEN SOS RESPONSE, MUTAGENESIS, AND RESISTANCE DURING THE RECOVERY PERIOD**

**Sreyashi Ghosh^1^, and Mehmet A. Orman^1^***

^1^William A. Brookshire Department of Chemical and Biomolecular Engineering, University of Houston, Houston, TX, USA

*Correspondence to: S222 Engineering Bldg 1, 4226 Martin Luther King Boulevard, Houston, TX 77204-4004, Phone: 713-743-6785, Email: [morman@central.uh.edu](mailto:morman@central.uh.edu)

**Supplementary figures**


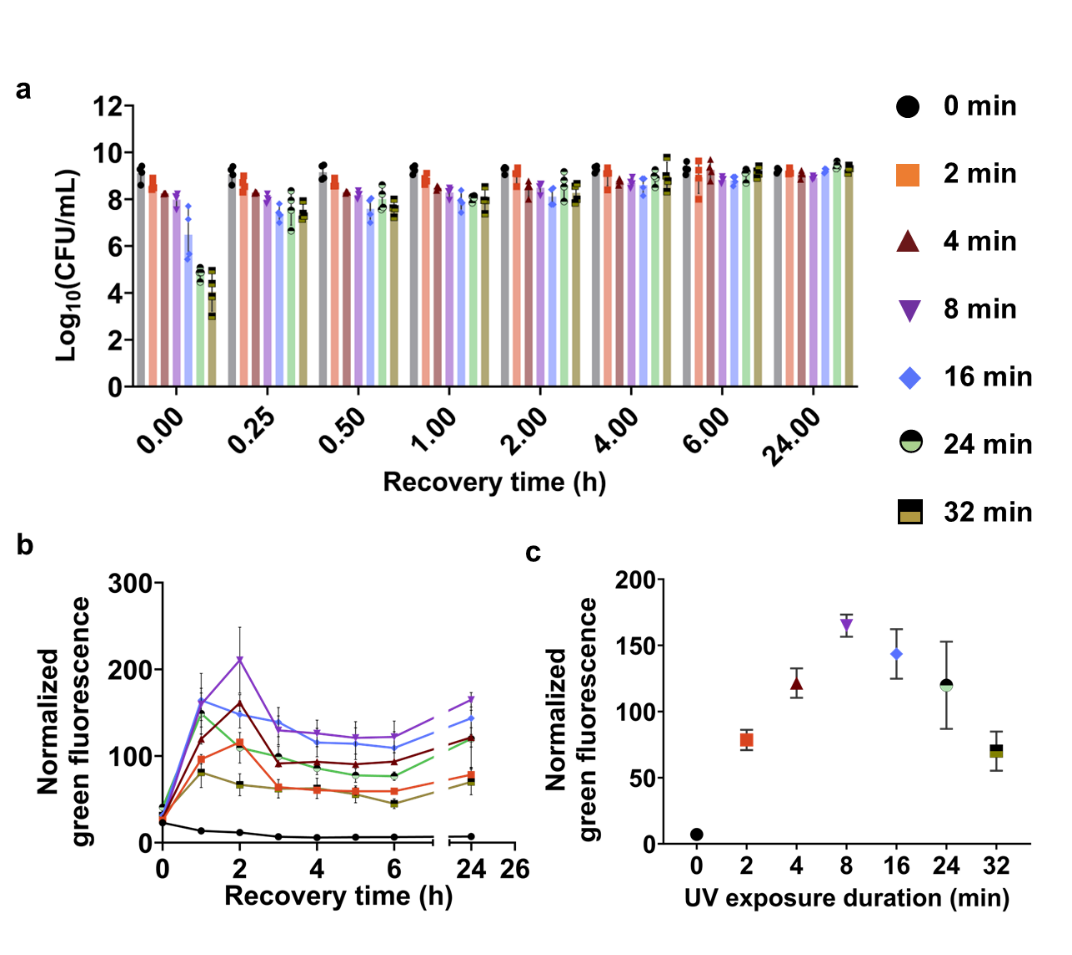


**Fig. S1: Quantifying colony forming units and *recA* expression levels after UV treatment.** (a) Mid-exponential-phase *E. coli* MG1655 pUA66- P*_recA_-gfp* cells were exposed to UV for 0, 2, 4, 8, 16, 24 and 32 min and recovered for 24 h. At indicated time points (t = 0 h, 0.25 h, 0.5 h, 1 h, 2 h, 4 h, 6 h, and 24 h), cells were collected and spotted on agar plates to enumerate colony formation units (CFU). (b) Temporal profiles of P*_recA_-gfp* expression during the recovery period with varying durations of UV exposure, normalized with cell density (OD_600_) at corresponding time points. (c) Normalized P*_recA_-gfp* expression levels at 24 hours of recovery with varying durations of UV exposure. n=4. Data corresponding to each time point represents mean value ± standard deviation.


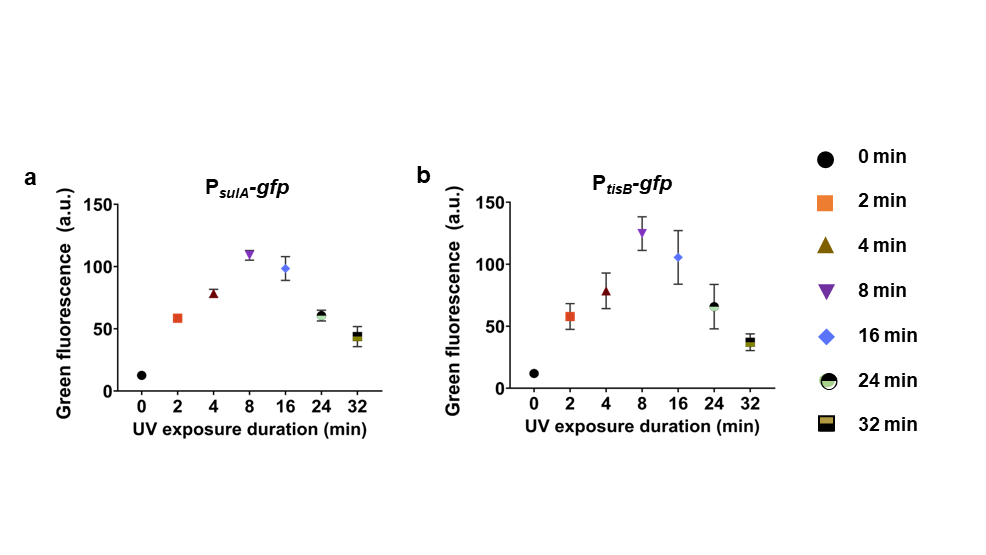


**Fig. S2: Profiles of P*_sulA_-gfp* and P*_tisB_-gfp* expression with varying UV exposure time after 24 h of recovery.** (a-b) Mid-exponential-phase *E. coli* MG1655 pUA66- P*_sulA_-gfp* and *E. coli* MG1655 pUA66- P*_tisB_-gfp* cells, respectively, were exposed to UV for 0, 2, 4, 8, 16, 24 and 32 min and recovered for 24 h. After 24 h of recovery, culture samples were collected to measure green fluorescence with a plate reader. n=4. Data corresponding to each time point represent mean value ± standard deviation.


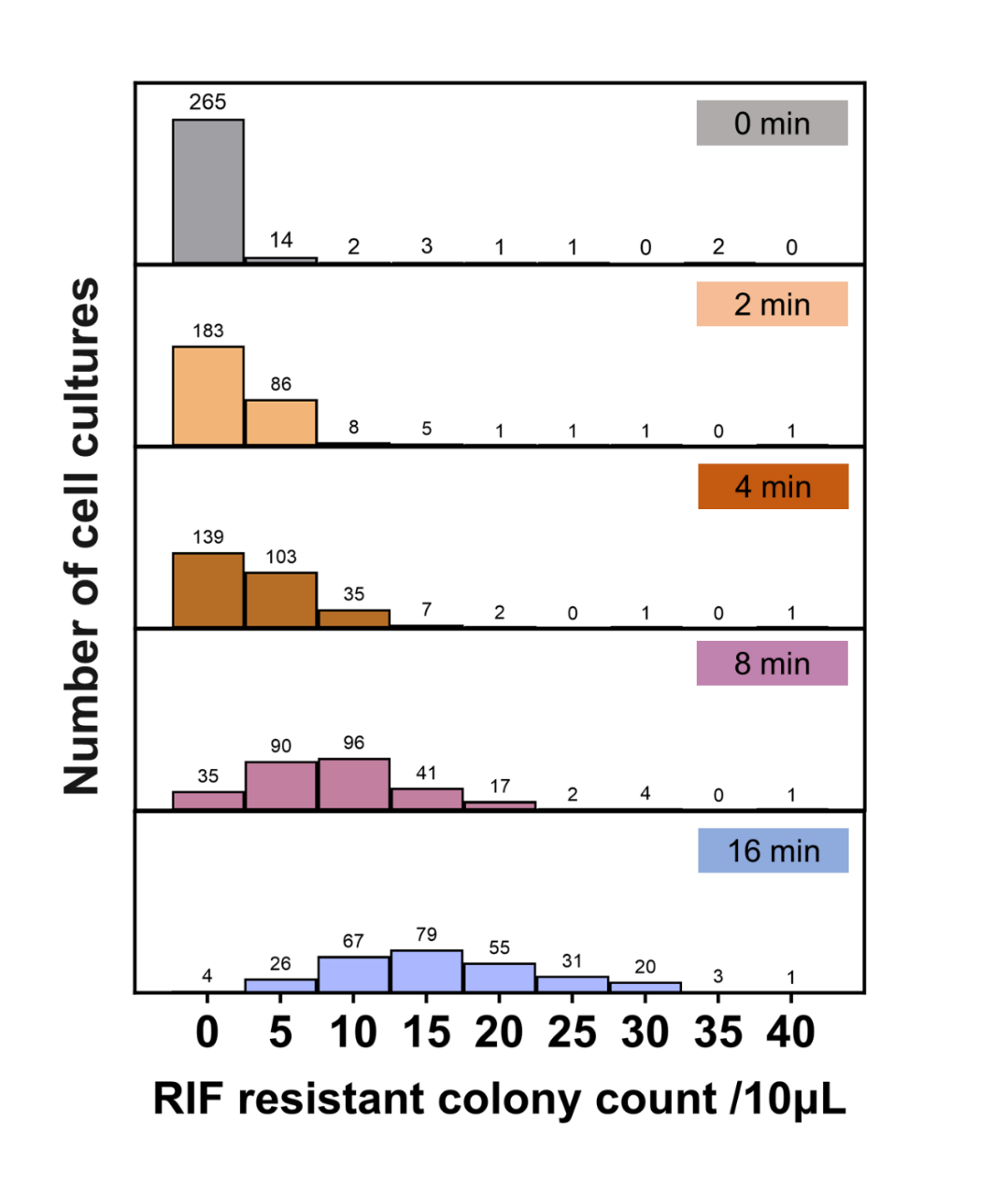


**Fig. S3: Validation of the correlation between the UV-induced SOS response and mutagenesis levels using a 96-well plate format.** A total of 288 independent cell cultures were exposed to UV for different durations. Subsequently, a 10 μL sample from each culture was plated on RIF agar plates after a 24-hour recovery period. The RIF-resistant colony data for each experimental condition were used to generate a frequency distribution, and a histogram plot was employed to derive the probability distribution of RIF-resistant colony generation across various durations of UV exposure.


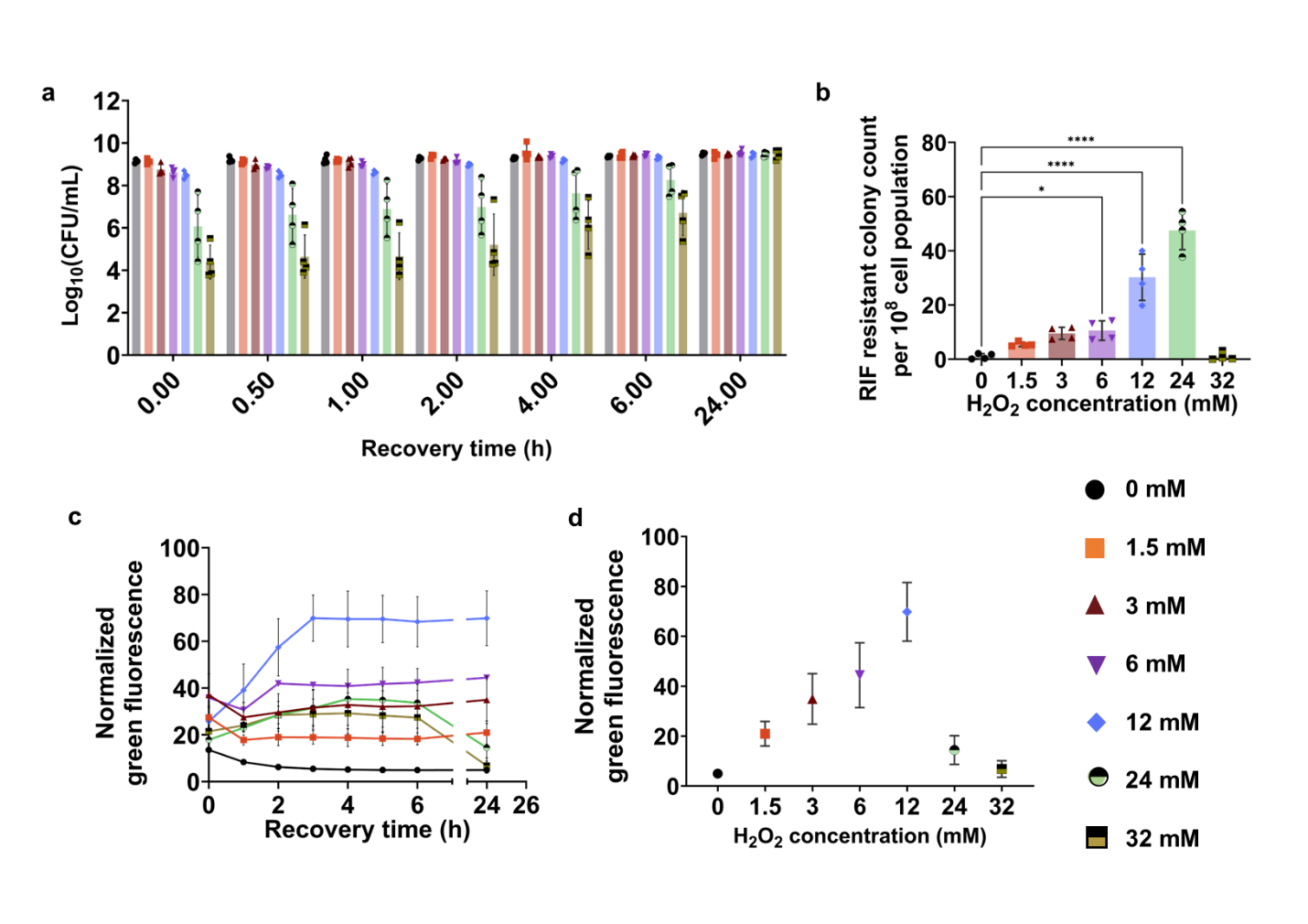


**Fig. S4: Correlation between hydrogen peroxide-induced SOS response and mutagenesis.** (a) Mid-exponential-phase *E. coli* MG1655 pUA66- P*_recA_-gfp* cells were exposed to hydrogen peroxide (H_2_O_2_) at varying concentrations (1.5 mM, 3mM, 6mM, 12mM, 24mM and 32mM) for 30 min and then washed to remove H_2_O_2_ followed by a recovery for 24 h. A sample with no H_2_O_2_ (0mM) was used as a control. At indicated time points during recovery (t = 0 h, 0.5 h, 1 h, 2 h, 4 h, 6 h and 24 h), cells were collected and spotted on agar plate to enumerate colony forming units (CFU). (b) Quantification of mutant cells reported as RIF resistant colony count per 10^8^ cell population for different H_2_O_2_ concentrations. *n* = 4. (c) Temporal profiles of P*_recA_-gfp* expression during the recovery period with varying H_2_O_2_ concentrations, normalized with cell density (OD_600_) at corresponding time points. (d) Normalized P*_recA_-gfp* expression levels at 24 hours of recovery with varying H_2_O_2_ concentrations. Statistical analysis was performed using one-way ANOVA with Dunnett’s post-test, where **P* < 0.05, *****P* < 0.0001. Data corresponding to each time point represent mean value ± standard deviation.


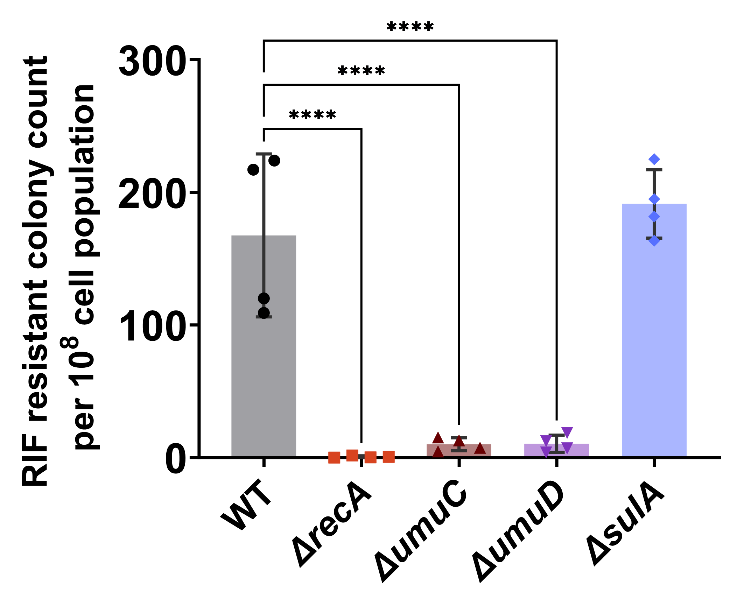


**Fig. S5: Quantifying UV-induced mutagenesis in *E. coli* K12 BW25113 WT, Δ*recA*, Δ*umuC*, Δ*umuD*, and Δ*sulA* strains.** Mid-exponential-phase *E. coli* K12 BW25113 WT, Δ*recA*, Δ*umuC*, Δ*umuD*, and Δ*sulA* cells were exposed to UV for 16 min and then allowed to recover for 24 hours. Following the recovery period, cells were plated on rifampicin-agar plates to quantify the extent of mutagenesis. n=4. Statistical analysis was performed using one-way ANOVA with Dunnett’s post-test, where *****P* < 0.0001. Data corresponding to each time point represent mean value ± standard deviation.


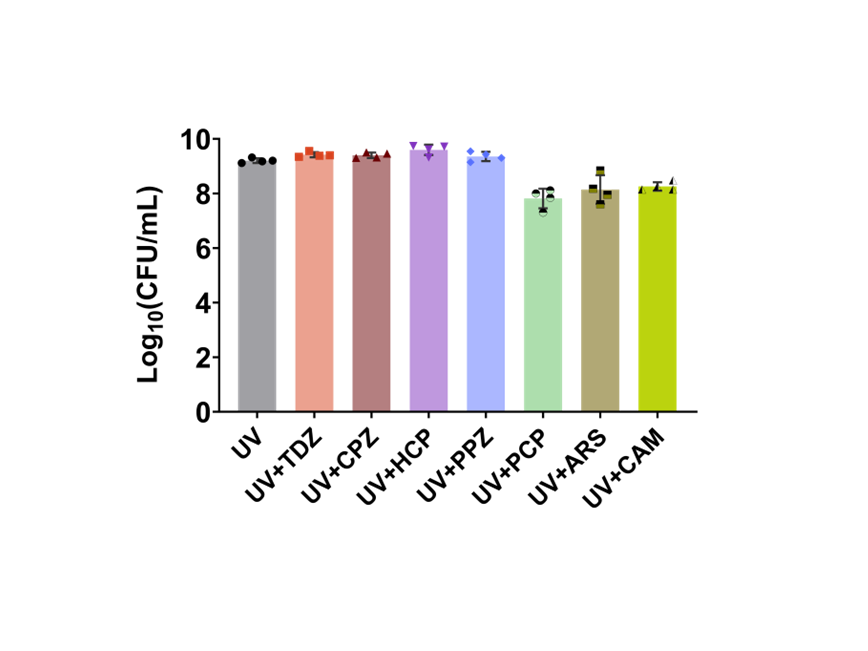


**Fig. S6: Quantifying colony forming units after recovery from UV and chemical inhibitors.** Mid-exponential phase *E. coli* MG1655 pUA66- P*_recA_-gfp* cells were treated with chemical inhibitors: thioridazine (TDZ; 1mM)​​, chlorpromazine​ (CPZ; 0.25mM), hexachlorophene (HCP; 0.1 mM), perphenazine (PPZ; 1mM), arsenate (ARS; 1mM), pentachlorophenol ​(PCP; 0.3 mM), chloramphenicol (CHL; 20 μg/mL). Then, cultures were exposed to UV radiation for 16 min and recovered for 24 h. After recovery, cells were collected, washed to remove chemicals, and spotted on agar medium to enumerate colony-forming units (CFU). n=4. Data corresponding to each time point represent mean value ± standard deviation.


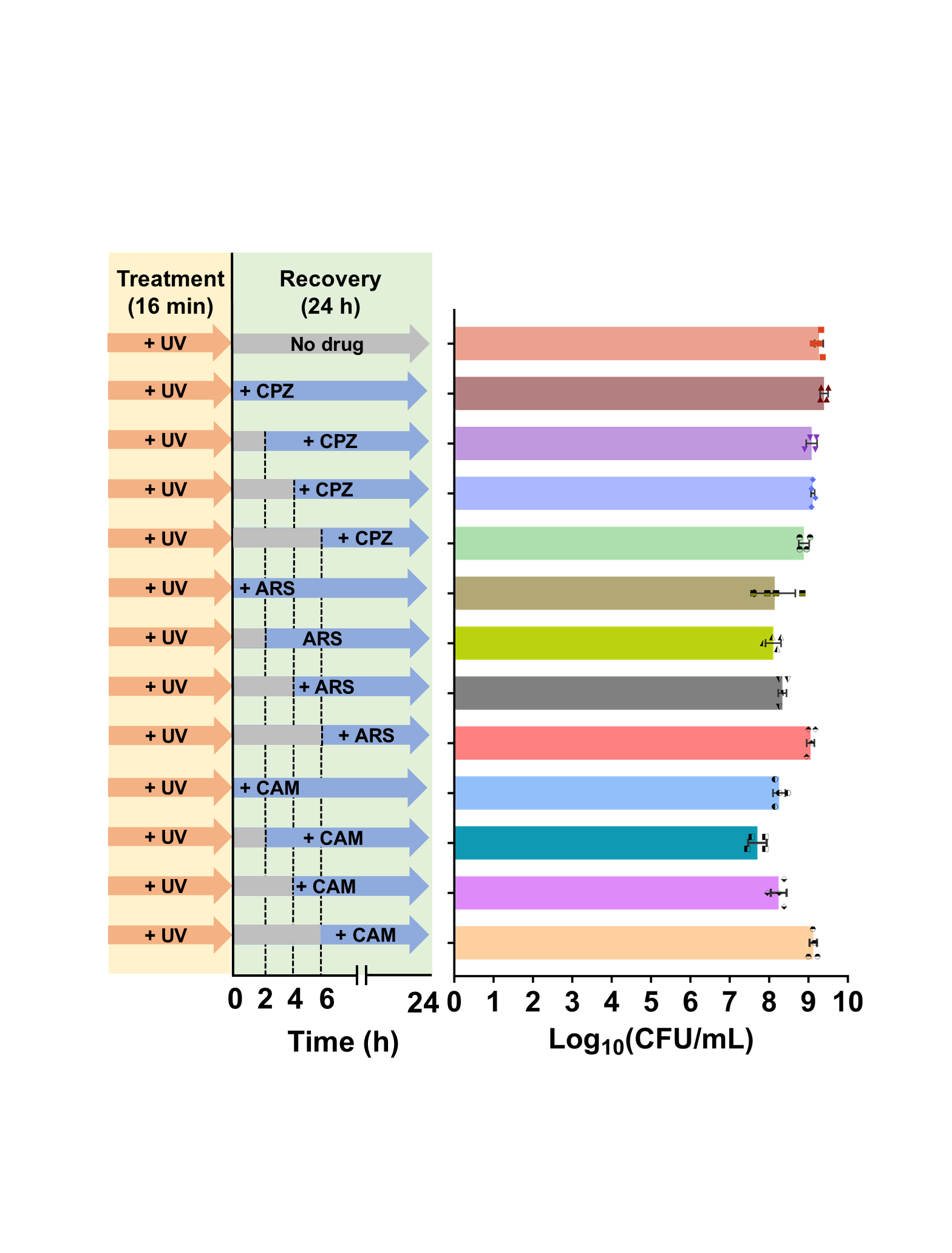


**Fig. S7:** **Quantification of colony-forming units in cell cultures treated with chemical inhibitors at various time points during recovery following UV treatment.** Mid-exponential phase *E. coli* MG1655 cells were exposed to UV for 16 min and the metabolic inhibitors, chlorpromazine​ (CPZ; 0.25mM), arsenate (ARS; 1mM), and chloramphenicol (CAM; 20 μg/mL), were added at the indicated time points (as shown in the schematics) during the recovery (0, 2, 4 and 6 h). After 24 h of recovery, CFU levels were quantified by washing the recovered samples and then plating them on the LB agar medium. n=4. Data corresponding to each time point represents mean value ± standard deviation.


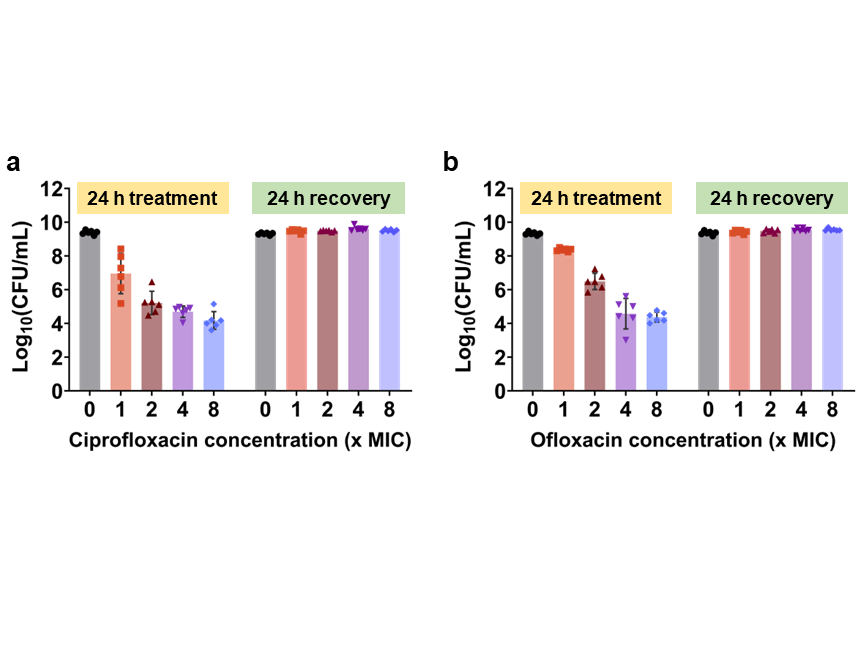


**Fig. S8: Quantifying colony forming units after 24 h fluoroquinolone treatment and 24 h recovery.** Mid-exponential phase *E. coli* MG1655 cell cultures with an average cell density of 7x10^8^ CFUs/ml were treated with (a) ciprofloxacin (CFX; MIC: 0.02 μg/mL) and (b) ofloxacin (OFX; MIC: 0.07 μg/mL) at indicated concentrations for 24 h, then washed and recovered for 24 h in fresh media. After 24 h of recovery, cells were washed and plated to enumerate colony-forming units (CFU). n=6. Data corresponding to each time point represent mean value ± standard deviation.


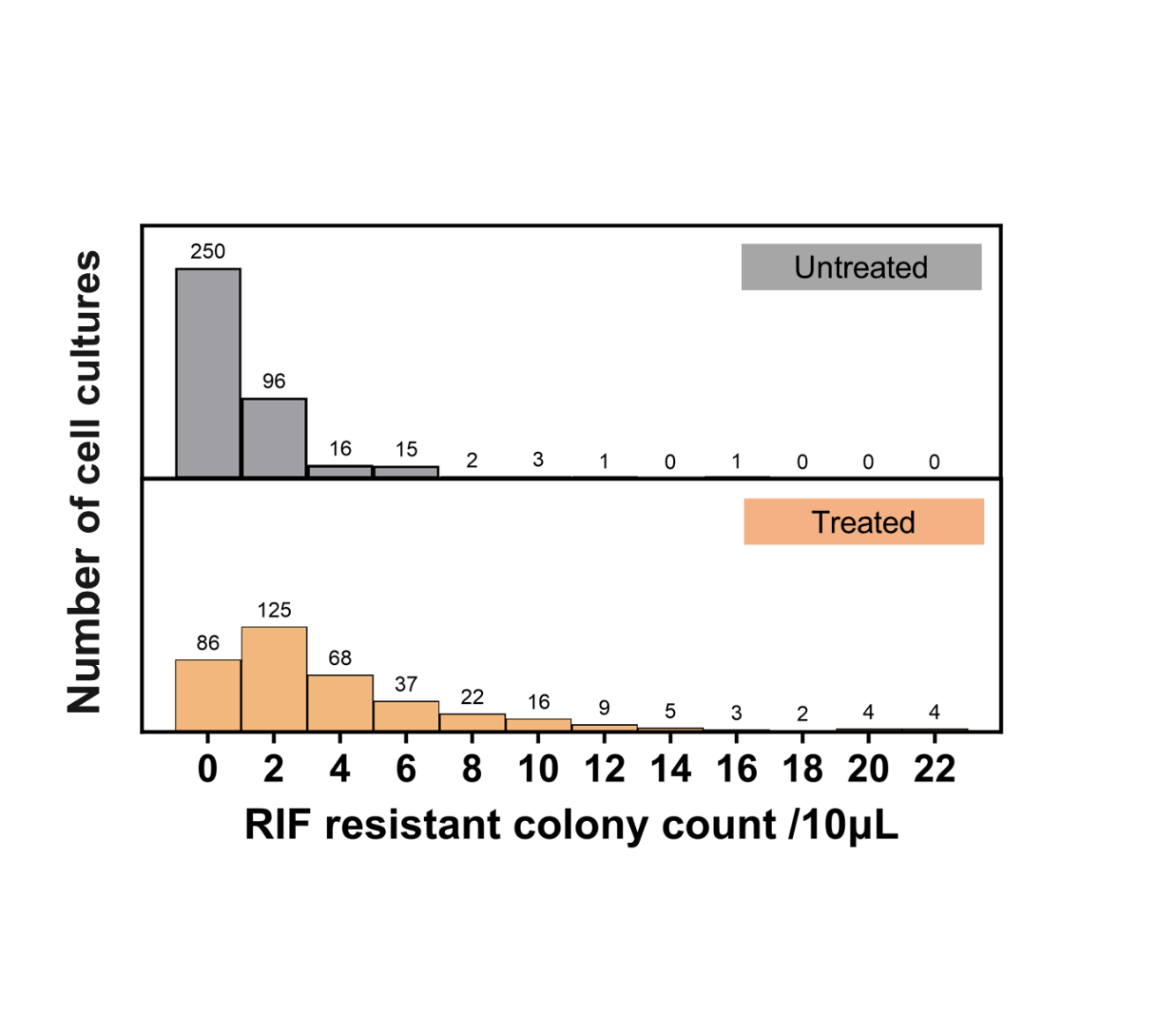


**Fig. S9: Validation of the correlation between the ciprofloxacin-induced SOS response and mutagenesis levels using a 96-well plate format.** A total of 384 distinct cell cultures were subjected to treatment with 1x MIC of CFX for 24 hours, followed by a 24-hour recovery in antibiotic-free fresh media. A parallel process was applied to the untreated control, utilizing solvent instead of CFX. Following the recovery period, a 10-μL sample from each culture was plated onto RIF agar plates. A frequency distribution was generated from the RIF-resistant colony data for the two experimental conditions, and a histogram plot was employed to derive the probability distribution.


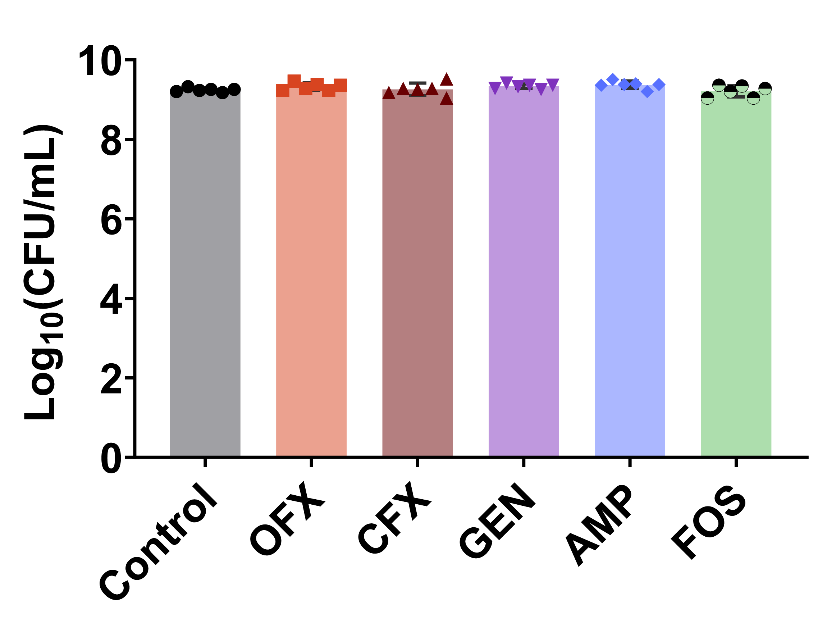


**Fig. S10: Quantifying colony forming units after recovery from conventional antibiotics.** Mid-exponential phase *E. coli* MG1655 pUA66- *P_recA_-gfp* cells were treated with 1x MIC of conventional antibiotics: ciprofloxacin (CFX; MIC: 0.02 μg/mL), ofloxacin (OFX; MIC: 0.07 μg/mL), gentamycin (GEN; MIC: 0.5 μg/mL ), ampicillin (AMP; MIC: 6 μg/mL), and fosfomycin (FOS; MIC: 0.1 μg/mL) for 24 h, and, then, washed and recovered for 24 h in fresh media. After 24 h of recovery, cells were washed and plated to enumerate colony-forming units (CFU). n=6. Data corresponding to each time point represent mean value ± standard deviation.


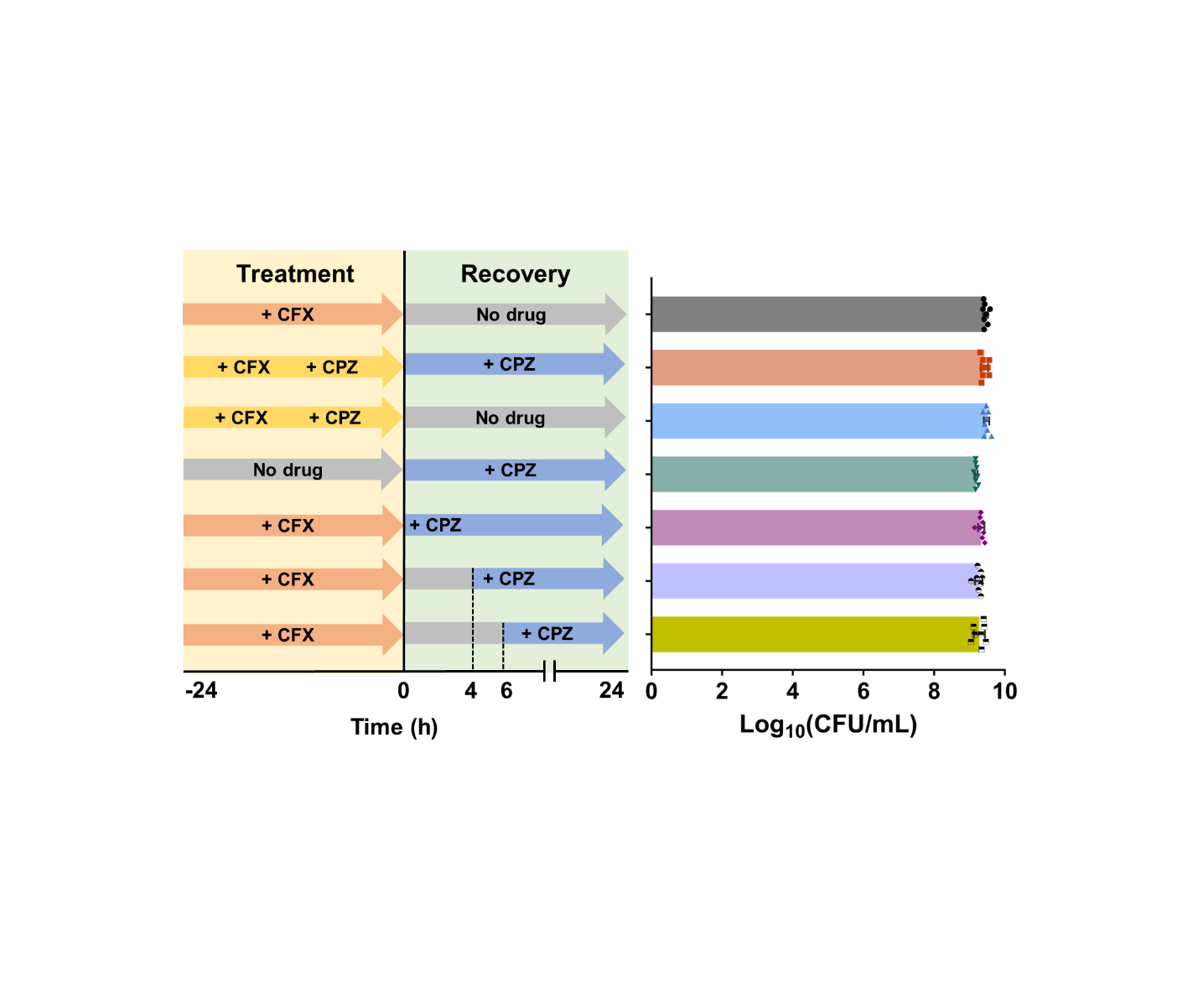


**Fig. S11: Quantification of colony-forming units in cell cultures treated with ciprofloxacin and/or chlorpromazine at various time points.** *E. coli* cells were treated with CFX (1 x MIC) and CPZ (0.25 mM) following the experimental schematics shown on the panel. After 24 h of recovery, CFU levels were quantified by washing the recovered samples and then plating them on the LB agar medium. n=8. Data corresponding to each time point represents mean value ± standard deviation.

**Supplementary tables**

**Table S1: Bacteria strains and plasmids used in this study**

| **Bacterial Strains** | **Source** |
| --- | --- |
| *Escherichia coli* K-12 MG1655 Wild Type | Gift from Dr. Mark P. Brynildsen |
| *Escherichia coli* K-12 MG1655 Δ*recA* | Previous study |
| *Escherichia coli* K-12 BW25113 WT | Keio Knockout Collection |
| *Escherichia coli* K-12 BW25113 Δ*recA* | Keio Knockout Collection |
| *Escherichia coli* K-12 MG1655 Δ*sulA* | Keio Knockout Collection |
| *Escherichia coli* K-12 MG1655 Δ*umuC* | Keio Knockout Collection |
| *Escherichia coli* K-12 MG1655 Δ*umuD* | Keio Knockout Collection |
| *Klebsiella pneumoniae* CXY 130 | Gift from Dr. Kevin W. Garey |
| *Acinetobacter baumannii* BAA-1605 | Gift from Dr. Kevin W. Garey |
|  |  |
| **Bacterial Plasmids** | **Source or Reference** |
| pUA66 P*recA-gfp* | Gift from Dr. Mark P. Brynildsen |
| pUA66 P*sulA*-*gfp* | Gift from Dr. Mark P. Brynildsen |
| pUA66 P*tisB-gfp* | Gift from Dr. Mark P. Brynildsen |

**Table S2: Antibiotics used in this study**

| **Bacteria name** | **Drug name** | **Minimum inhibitory concentration (MIC)** | **Concentration used in this study** |
| --- | --- | --- | --- |
| *Escherichia coli* (MG1655) | Ofloxacin  (OFX) | 0.07 μg/mL | 0.07, 0.14, 0.28, 0.56 μg/mL |
|  | Ciprofloxacin (CFX) | 0.02 μg/mL | 0.02, 0.04, 0.08, 0.16 μg/mL |
|  | Ampicillin  (AMP) | 6 μg/mL | 6 μg/mL |
|  | Fosfomycin  (FOS) | 0.1 μg/mL | 0.1 μg/mL |
|  | Gentamycin  (GEN) | 0.5 μg/mL | 0.5 μg/mL |
| *Klebsiella pneumoniae* (CXY130) | Ciprofloxacin (CFX) | 3.5 μg/mL | 3.5 μg/mL |
| *Acinetobacter baumannii* (BAA-1605) | Ciprofloxacin (CFX) | >32 μg/mL | - |
